# Unrecognized maternal heart rate artefact in cases of perinatal mortality reported to the United States Food and Drug Administration from 2009 to 2019: a critical patient safety issue

**DOI:** 10.1186/s12884-019-2660-5

**Published:** 2019-12-16

**Authors:** Daniel J. Kiely, Lawrence W. Oppenheimer, James C. Dornan

**Affiliations:** 1 Department of Obstetrics and Gynecology, Hôpital de Thetford Mines, 1717 rue Notre Dame Est, Thetford Mines, Québec, G6G 2V4 Canada; 2 Department of Obstetrics and Gynecology, Division of Maternal-Fetal Medicine, Faculty of Medicine, University of Ottawa, The Ottawa Hospital, 501 Smyth Road, Box 804, Ottawa, Ontario K1H 8L6 Canada; 3 Department of Fetal Medicine, Department of Obstetrics and Gynecology, Faculty of Medicine, Queens University Belfast (rtrd) and Chair Health and Life Sciences, Ulster University, York Street, Belfast, County Antrim, Belfast, Northern Ireland BT15 1ED

**Keywords:** Fetal heart rate, Cardiotocography, Perinatal death, Stillbirth, Brain hypoxia-ischemia, Product recalls and withdrawals, Pregnancy, Obstetrics, Fetal distress, Fetal monitoring

## Abstract

**Background:**

Maternal heart rate artefact is a signal processing error whereby the fetal heart rate is masked by the maternal pulse, potentially leading to danger by failure to recognize an abnormal fetal heart rate or a pre-existing fetal death. Maternal heart rate artefact may be exacerbated by autocorrelation algorithms in modern fetal monitors due to smooth transitions between maternal and fetal heart rates rather than breaks in the tracing. In response, manufacturers of cardiotocography monitors recommend verifying fetal life prior to monitoring and have developed safeguards including signal ambiguity detection technologies to simultaneously and continuously monitor the maternal and fetal heart rates. However, these safeguards are not emphasized in current cardiotocography clinical practice guidelines, potentially leading to a patient safety gap.

**Methods:**

The United States Food and Drug Administration Manufacturer and User Facility Device Experience database was reviewed for records with event type “Death” for the time period March 31, 2009 to March 31, 2019, in combination with search terms selected to capture all cases reported involving cardiotocography devices. Records were reviewed to determine whether maternal heart rate artefact was probable and/or whether the report contained a recommendation from the device manufacturer regarding maternal heart rate artefact.

**Results:**

Forty-seven cases of perinatal mortality were identified with probable maternal heart rate artefact including 14 with antepartum fetal death prior to initiation of cardiotocography, 14 with intrapartum fetal death or neonatal death after initiation of cardiotocography, and 19 where the temporal relationship between initiation of cardiotocography and death cannot be definitively established from the report. In 29 cases, there was a recommendation from the manufacturer regarding diagnosis and/or management of maternal heart rate artefact.

**Conclusions:**

This case series indicates a recurring problem with undetected maternal heart rate artefact leading to perinatal mortality and, in cases of pre-existing fetal death, healthcare provider confusion. In response, manufacturers frequently recommend safeguards which are found in their device’s instructions for use but not in major intrapartum cardiotocography guidelines. Cardiotocography guidelines should be updated to include the latest safeguards against the risks of maternal heart rate artefact. An additional file summarizing key points for clinicians is included.

## Background

Cardiotocography (CTG) is an imperfect technology but one of critical importance, as it may be a deciding factor in protecting against injury or death to the fetus during the birthing process. A potential pitfall in cardiotocography is artefact. There are two main classes of CTG artefact: maternal heart rate artefact and fetal heart rate artefact. Maternal heart rate artefact (MHRA) occurs when the maternal heart rate is captured as the input and the CTG monitor mistakenly outputs it as the fetal heart rate [1–4]. MHRA is particularly dangerous when an abnormal fetal heart rate is masked by a maternal heart rate which lies within the normal fetal range. Fetal heart rate artefact (FHRA) occurs when the signal input is from a fetus but the output is inaccurate. We define Type I FHRA as occurring when the input signal is from the intended fetus but there is doubling of the fetal heart rate, halving, or other signal processing errors. In contrast, Type 2 FHRA occurs exclusively in multiple gestation, when the input signal is captured from a fetus other than that intended (eg. Twin B) and the fetal heart monitor outputs this as the presumed fetal heart rate signal of the intended fetus (eg. Twin A). Signal ambiguity is defined as a specific cardiotocography artefact where the output (presumed fetal heart rate) is inaccurate because it is mistakenly derived from the maternal heart rate and/or, in multiple gestation, from a fetus other than that intended [2]. Signal ambiguity includes MHRA and Type 2 FHRA. Although MHRA and FHRA have been described in the obstetric literature for quite some time, what may be less appreciated is that the introduction of autocorrelation with more modern fetal monitors may lead to subtle and perhaps more frequent instances of signal ambiguity with smoother transitions (from maternal to fetal and vice versa and/or from one fetus to another in multiple gestation), as opposed to breaks in the tracing with older monitors [3, 5].

In response to concerns about signal ambiguity, manufacturers of fetal monitors developed what we have called signal ambiguity detection technologies. For example, Philips developed “cross-channel verification” and General Electric developed “heartbeat coincidence detection” [6–9]. Signal ambiguity detection technologies were further refined following a class 2 recall of Philips Avalon fetal monitors involving major adverse outcomes related to a failure to detect MHRA and other CTG artefacts in 2009 [6, 10, 11]. Of note, recall in this sense did not mean market withdrawal but rather initiation of corrective action by Philips and class 2 indicates intermediate level of health hazard as per the United States Food and Drug Administration [12].

**Fig. 1:**
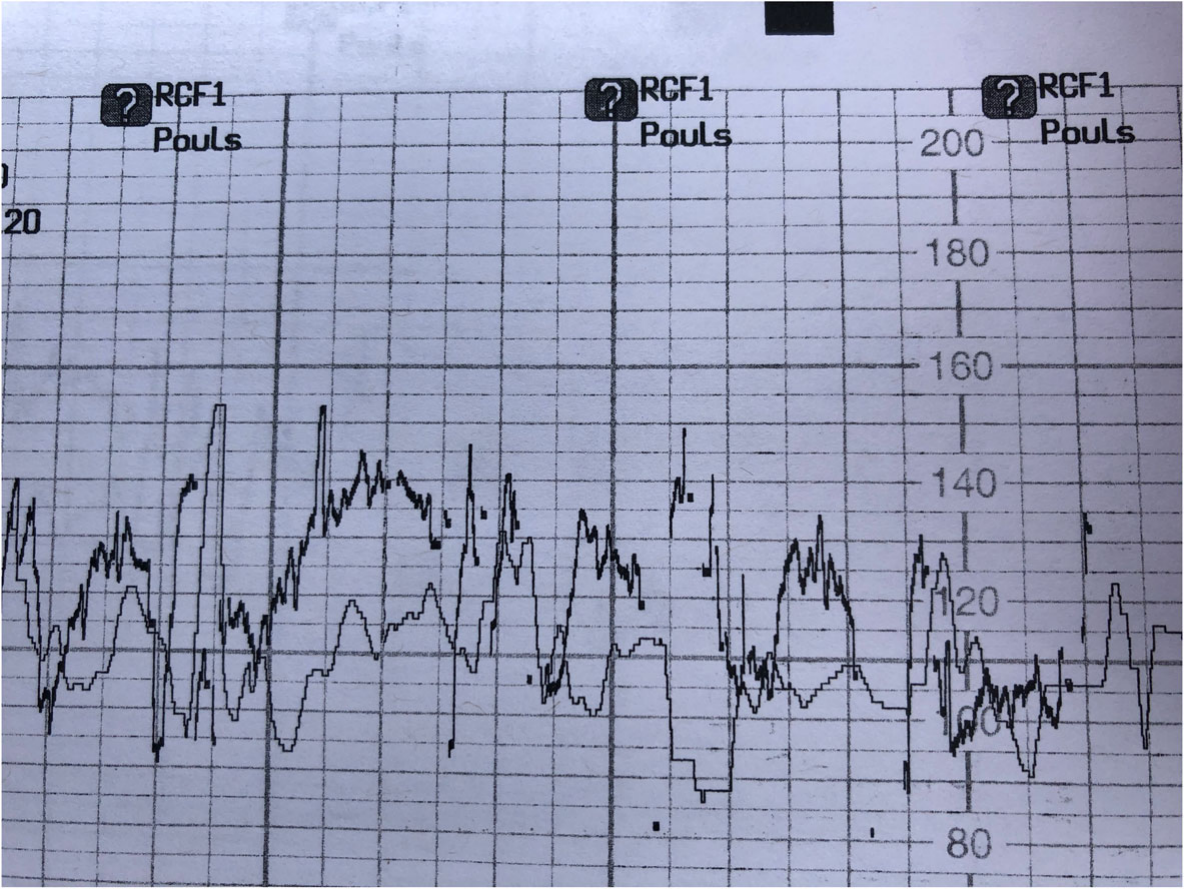
illustrates repeated coincidence alarms, indicated by the question marks with a Philips Avalon monitor**.** *This CTG monitor is formatted for the French language. With English formatting, the caption “RCF1, Pouls” beside the question mark icons would read “FHR1, Pulse”. The faint line is the presumed maternal heart rate and the dark line is the presumed fetal heart rate. Consent has been obtained from the patient to publish this image*

Signal ambiguity detection technologies involve continuously monitoring the maternal pulse and fetal heart rate (generally with both heart rates displayed on the monitor screen and the paper tracing), and emit “coincidence” alarms when the fetal and maternal heart rates are close enough together that one may, in fact, be from the same source [1, 3, 7–9]. These technologies can also be used to monitor twins simultaneously to detect FHRA Type 2. The coincidence alarm icon is two overlapping hearts for General Electric and a question mark for Philips (See Fig. 1) [3, 7, 9].

Another common recommendation from manufacturers is to verify fetal life by alternate means (such as directly palpation fetal movements or bedside ultrasound) prior to CTG monitoring in order to prevent undiagnosed fetal death [6–8].

We are concerned that there is a patient safety gap due to disconnect between manufacturers’ instructions for use and national/international CTG guidelines regarding signal ambiguity detection technology and CTG artefact more generally. This led us to review the United States Food and Drug Administration (FDA) Manufacturer and User Facility Device Experience (MAUDE) database: an open-access, online searchable databases of adverse event reports involving medical devices [13]. The FDA MAUDE database accepts reports from mandatory and voluntary reporters [14]. Voluntary reporters generally include health care professionals, patients, and families [14]. For our purposes, mandatory reporters include manufacturers and hospitals. As per the FDA, mandatory reporters are obligated to send reports to the FDA if they receive information suggesting that death or serious injury of a patient occurred due to a device that either: they marketed or that occurred in their facility [14]. Reports are accepted from all over the world. Reports from outside the United States of America are identified as “foreign”. Reports greater than 10 years old are archived and are no longer available through a simple online search of the database. FDA MAUDE database reports generally consist of two principal sections: the event description and the manufacturer narrative [13]. The event description is a summary of the event reported and the manufacturer’s narrative is the response and analysis of events from the firm which produces the device. The manufacturer’s narrative is sometimes in progress or absent.

Our aim was to assess the frequency of probable MHRA in cases of CTG-associated fetal and/or neonatal death in the US FDA MAUDE database and to assess if there are indications, in those reports, of gaps in healthcare provider education and training regarding strategies to decrease the risk of undetected MHRA. FHRA emerged as a topic in our analysis which we intend to explore further in future publications but was not our main focus here.

## Methods

The lead author, a community-based obstetrician-gynecologist, searched the US Food and Drug Administration (FDA) Manufacturer and User Facility Device Experience (MAUDE) database for records with event type “Death” for the time period March 31, 2009 to March 31, 2019, in combination with a series of search terms intended to capture all cases involving cardiotocography. Those search terms were: in brand name field: “fetal monitor”, “avalon”, “corometrics”, “fetal heart monitor”, “STAN S31”, “perinatal monitoring system”, “sonicaid”, “series 50”, “fetal/maternal monitor”, “maternal monitor”, “50 IP”, “huntleigh”, “S50 XM”, “50 XM”, “neoventa”, “obstetric data analyzer”, “fetal transducer”, “ultrasound transducer”, “ohmeda”, “coro 250”, “spacelabs”; and in the product code field: “HEL” (monitor, heart rate, fetal, ultrasonic), “HEK” (monitor, heart sound, fetal, ultrasonic), “HEI” (monitor, heart-valve movement, fetal, ultrasonic), “HGM” (system, monitoring, perinatal), “HFM” (fetal monitor), and “HEO” (fetal heart monitor). The search was initially conducted using product codes: “HEL”, “HEK”, “HEI” (as recommended in our communications with the US FDA) and brand name “fetal monitor” or “Avalon” (based on our exploratory searches) with subsequent searches conducted based on any new search terms related to CTG monitoring which we detected in an iterative fashion. The search was closed when we ceased detecting new search terms. Repeats, records not pertaining to fetal heart rate monitors, and records pertaining to central fetal monitoring systems were eliminated. What remained, we defined as reports of “cardiotocography-associated fetal and neonatal death”.

For each record retrieved, the lead author reviewed the following aspects: type of event (antepartum fetal death; intrapartum fetal death; neonatal death; cannot distinguish between antepartum and intrapartum fetal death; cannot distinguish between intrapartum fetal death and neonatal death; or cannot distinguish between antepartum fetal death, intrapartum fetal death, and neonatal death), likelihood that maternal heart rate artefact was probable, type of fetal monitor, and whether the manufacturer’s report is complete, in progress, or unknown. The criteria for determining a report contained probable maternal heart rate artefact and for determining whether there was a recommendation from the manufacturer regarding maternal heart rate artefact was pre-specified. Probable MHRA was defined to be present when the event description and/or manufacturer narrative included phrases or wording such as “confusion of maternal and fetal heart rate”, “coincidence”, identification of unexpected bradycardia after application of internal clip (fetal scalp electrode), or other words or phrases conveying that the author of the event description or manufacturer’s narrative believed MHRA was likely a factor. In cases where a fetus was already stillborn, we considered a positive “fetal” heart rate signal via the electronic fetal monitoring as evidence of probable MHRA. Additionally, we assessed whether each report contained a recommendation and/or suggestion from the manufacturer regarding diagnosis and/or management of maternal heart rate artefact. These recommendations and/or suggestions were pre-specified as follows: that the provider should have confirmed fetal life by alternate means prior to starting electronic fetal monitoring, that signal ambiguity detection technology should have been used, and/or that response to alarms from signal ambiguity detection technology should have been better.

The cases involving probable maternal heart rate artefact and/or those containing a recommendation from the manufacturer regarding maternal heart rate artefact were independently reviewed by two specialists in maternal fetal medicine using the same pre-specified criteria. Disagreements were resolved by consensus. There was no prior working relationship between the authors except that the first and third author had both been consulted as experts regarding one case of perinatal mortality and had some prior correspondence regarding that case. No future working relationship between the authors is planned except for possible future research collaboration and all work in separate jurisdictions with different medical licensing colleges. Of three experts approached by the lead author to collaborate on this research, the authors are the only ones who agreed to review the data. One declined prior to data review. Collaborators were invited to join this research project for the following reasons: LO demonstrated expertise in MHRA by publication [4], and JD, demonstrated expertise in MHRA by the case review mentioned earlier. The first author had previously published on MHRA and the Philips Avalon fetal monitor recall [15–18].

Reports are all openly available online through the FDA MAUDE database, although they will become more difficult to access over time as reports greater than 10 years old are archived. In order to strengthen our methodology, all reports reviewed here have been archived through the Figshare digital repository [19].

## Results

A total of 500 reports were obtained by the search strategy and reviewed. After eliminating repeats, reports not relevant to fetal heart rate monitors, and 4 cases involving central fetal monitors, we were left which 117 reports which form our core dataset. The source is listed as “foreign” in 35 of those reports. All the 117 FDA reports in our dataset are available in the open access Figshare digital repository under the project title “Cardiotocography-associated fetal and neonatal deaths reported to the US FDA 2009-2019” [19]. We identified 47 cases out of the total of 117 (40%) with probable maternal heart rate artefact (these 47 cases are available in abbreviated form in Additional file 1 and in their entirety in a folder of the Figshare repository) [20]. The manufacturer’s report frequently suggests additional steps should have been taken to mitigate the risks of undetected maternal heart rate artefact: confirmation of fetal life by an alternate means prior to cardiotocography (16 cases), activate signal ambiguity detection technology (16 cases), and/or improve response to alarms from signal ambiguity technology (10 cases), for a total of 29 cases out of the total 117 (25%) with at least one of these recommendations (these 29 cases are available in abbreviated form in Additional file 2 and in their entirety in a folder of the Figshare repository) [21]. There were reports from a variety of different fetal monitors (Table 1).

**Table 1:** Type of fetal monitor in cases of fetal or neonatal death associated with cardiotocography reported to the U.S. FDA, March 31, 2009 to March 31, 2019

Type of fetal monitor	Cases out of total 117 cases	Cases out of 47 with probable MHRA (% of the cases with probable MHRA)
Avalon (Philips)	72 (62%)	34 (72%)
Corometrics (General Electric)	9 (8%)	2 (4%)
HP 1350 (Hewlett Packard)	1 (0.8%)	0 (0%)
SONICAID (HUNTLEIGH)	9 (8%)	4 (9%)
Neoventa STAN	4 (3%)	0 (0%)
50 Series Philips	20 (17%)	7 (15%)
Spacelabs	2 (0.02%)	0 (0%)

We then analyzed the 47 cases with probable maternal heart rate artefact (Table 2). Again, we found a substantial number of reports where there was a recommendation or suggestion from the manufacturer that additional steps should have been taken: verify the fetus is alive prior to CTG monitoring (14 reports), activate signal ambiguity technology (14 cases), improve response to signal ambiguity detection technology alarms (10 cases), for a total of 25 cases out of 47 (53%) with at least one of these three recommendations. This is less than our result of 29 cases with such a recommendation from the whole series because there were 4 cases where there was a recommendation regarding MHRA but where the description of the event was not sufficient for us to conclude it involved probable MHRA.

**Table 2:** Cases of fetal or neonatal death associated with cardiotocography reported to the U.S. FDA, March 31, 2009 to March 31, 2019; subset where reports indicate probable maternal heart rate artefact

Event type	Number of cases (% of all 47 cases)	Recommendation to verify fetal life by alternate means prior to cardiotocography (% of cases of that event type)	Recommendation to use signal ambiguity detection technology (% of cases of that event type)	Recommendation to improve response to alarms from signal ambiguity detection technology (% of cases of that event type)	Total cases with recommendation to verify fetal life by alternate means prior to cardiotocography, use signal ambiguity detection technology, or respond to alarms from such technology. (% of case of that event type)
Antepartum fetal death	14 (30%)	9 (60%)	6 (40%)	1 (7%)	9 (60%)
Cannot distinguish antepartum from intrapartum fetal death	17 (36%)	5 (29%)	4 (24%)	0 (0%)	6 (35%)
Cannot distinguish between antepartum fetal death, intrapartum fetal death, or neonatal death	2 (4%)	0 (0%)	0 (0%)	1 (50%)	1 (50%)
Intrapartum fetal death	3 (6%)	0 (0%)	1 (33%)	2 (67%)	2 (67%)
Cannot distinguish intrapartum fetal death from neonatal death	3 (6%)	0 (0%)	1 (33%)	3 (100%)	3 (100%)
Neonatal death	8 (17%)	0 (0%)	2 (25%)	3 (38%)	4 (50%)
Total	47	14 (29%)	14 (29%)	10 (20%)	25 (53%)

We separated the cases with probable maternal heart rate into those where either: one, there was an antepartum fetal death or a possible antepartum fetal death prior to initiation of CTG monitoring or two, it appeared relatively certain that there was an intrapartum fetal death or neonatal death after initiation of CTG monitoring. In cases of antepartum fetal death, MHRA may lead to unnecessary cesarean, healthcare provider team confusion, and emotional distress for the patient. However, in cases of intrapartum fetal death or neonatal death, MHRA is a potential direct contributor to fetal/neonatal death by masking fetal distress and potentially precluding more timely intervention. In the 33 cases of probable MHRA involving or possibly involving antepartum fetal death, the manufacturer’s report recommends verification of fetal life prior to CTG monitoring in 14 cases (42%), activating signal ambiguity detection technology in 10 cases (30%), and better response to signal ambiguity detection technology in 2 cases (6%) for a total of 16 cases with one of these recommendations (48%). In the 14 cases of probable MHRA involving intrapartum fetal death or neonatal death (available in abbreviated form in Additional file 3 and, in their entirety, in a folder of the Figshare repository) [22], there are no recommendations to verify fetal life prior to CTG monitoring (as would be expected as these were cases where the fetus is presumed to have been alive at the start of CTG monitoring), 4 cases with a recommendation to use signal ambiguity technology (29%), and 8 cases with a recommendation to improve response to alarms from signal ambiguity technology (57%), for a total of 9 cases (64%) with one of these recommendations.

We have abstracted the salient fields of every report which we judged to include probable MHRA and/or a recommendation regarding MHRA from the manufacturer into compact documents which we have included as additional files. The fields we included are fetal monitor type, event date if available, manufacturer narrative (full), event description (full), MDR report key, and report number. There are 3 such compact files: one including all cases of probable MHRA (Additional file 1), one including all cases involving a recommendation from the manufacturer involving MHRA (Additional file 2), and one involving all cases of where the event type is clearly intrapartum death or neonatal death (as opposed to cases of antepartum or possible antepartum fetal death) with probable MHRA (Additional file 3). Further, in all compact files, the relevant sections which led to inclusion of the report are highlighted in yellow and sections summarizing the event are highlighted in green. This allows an interested reader to review all reports in a short amount of time and decide for themselves regarding the strength of our conclusions.

From the 14 cases of neonatal death or intrapartum fetal death involving probable MHRA, we provide a few excerpts here from the manufacturers’ reports in order to give a sense of the actual clinical problems at play. In one case of neonatal death: “The logs of the monitor showed coincidence alarms at the time of the reported incident which were silenced manually by the user” [22, 23]. In a case of neonatal death from hypoxic ischemic encephalopathy: “The maternal heart rate was not monitored … .thus no coincidence notation was possible … At … .the trace shows less decelerations and normal variability … This would be an unlikely, sudden clinical improvement of the fetus and is probably caused by the ultrasound switching to the prominent pulse source of the mother.” [22, 24] From a case of intrapartum fetal death: “From … .onwards, there is no safe recording of the child (fetus) anymore … Coincidence alarms were reported correctly and appear on the traces regularly and repetitively” [22, 25]. From a case of neonatal death: “after … .the ultrasound almost continuously records a maternal signal. CCV (cross-channel verification) warning is given repeatedly” [22, 26]. From another case of neonatal death: “the maternal heart rate (mhr) increased and coincided with the fetal heart rate (fhr), however, the fetal monitor showed/printed question marks … the question marks were either ignored or not correctly interpreted due to human error” [22, 27]. From a case of neonatal death, “From that moment onwards, the fhr (fetal heart rate) trace rarely shows signals from the fetus, and is instead almost exclusively showing a maternal signal … .the fetal monitor issues multiple coincidence alerts” [22, 28]. From a case of intrapartum fetal death: “at some point hours before delivery, his fetal heart rate became distressed. Instead of picking up this distress, the … fetal heart rate monitor made a smooth transition to the maternal heart rate, confusing the healthcare providers into believing the baby’s heart rate was fine … the strip chart indicated the maternal heart rate and fetal heart rate actually overlapped 5 times during the monitoring session, as shown by the hbc (heartbeat coincidence) indication …. Another member of the ob team communicated they didn’t know about overlapping heartbeats and, therefore, did not understand the indications. The obstetricians communicated they did not read the monitor user’s manual and did not understand heartbeat coincidence.” [22, 29]

## Discussion

The case series reported here suggests an ongoing concern with MHRA in cases of cardiotocography-associated fetal and neonatal death reported to the FDA between 2009 and 2019. The case series reported here is, to the best of our knowledge, larger than prior case reports and case series of perinatal mortality involving MHRA, most of which involve only one to two cases of fetal/neonatal death [2, 30–33]. This case series is also more contemporary, involving more CTG monitors with autocorrelation and signal ambiguity detection technologies, and is the first, to our knowledge, to bring to light the manufacturer’s response to such reports. We note that manufacturer recommendations (confirm fetal life by alternate means prior to CTG monitoring, utilize signal ambiguity technology, and/or better respond to alarms from signal ambiguity detection technologies) are not emphasized, if mentioned at all, in the intrapartum fetal monitoring/cardiotocography guidelines which we have reviewed [34–41].

**Fig. 2:**
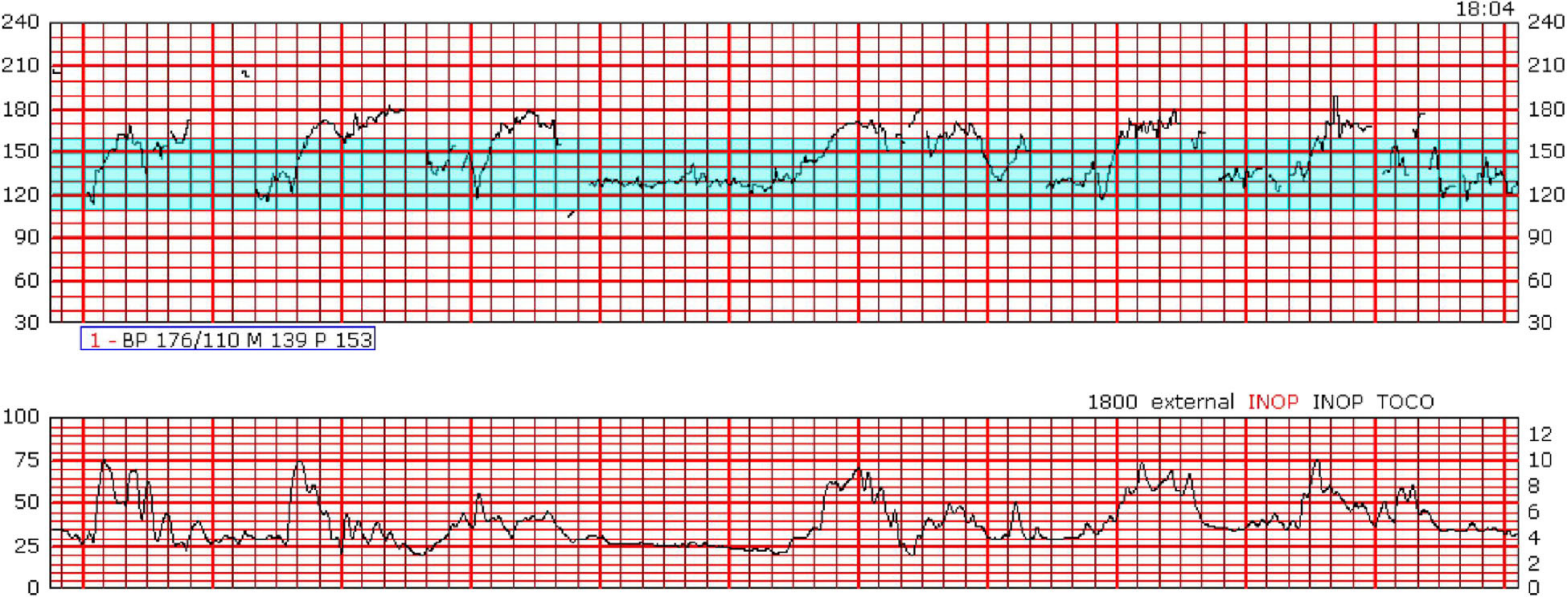
CTG tracing immediately prior to delivery of a stillborn infant**.** *CTG tracings in early labor were normal and it seems likely that the CTG monitoring switched from recording the fetal heart rate to recording the maternal heart rate, although the exact transition point is difficult to determine. Reprinted from American Journal of Obstetrics and Gynecology,* https://www.sciencedirect.com/journal/american-journal-of-obstetrics-and-gynecology*, Vol 198, Issue 6, June 2008, Pages 717–24, Neilson DR, Freeman RK, Mangan S. Signal ambiguity resulting in unexpected outcome with external fetal heart rate monitoring.© 2008, with permission from Elsevier*

**Fig. 3:**
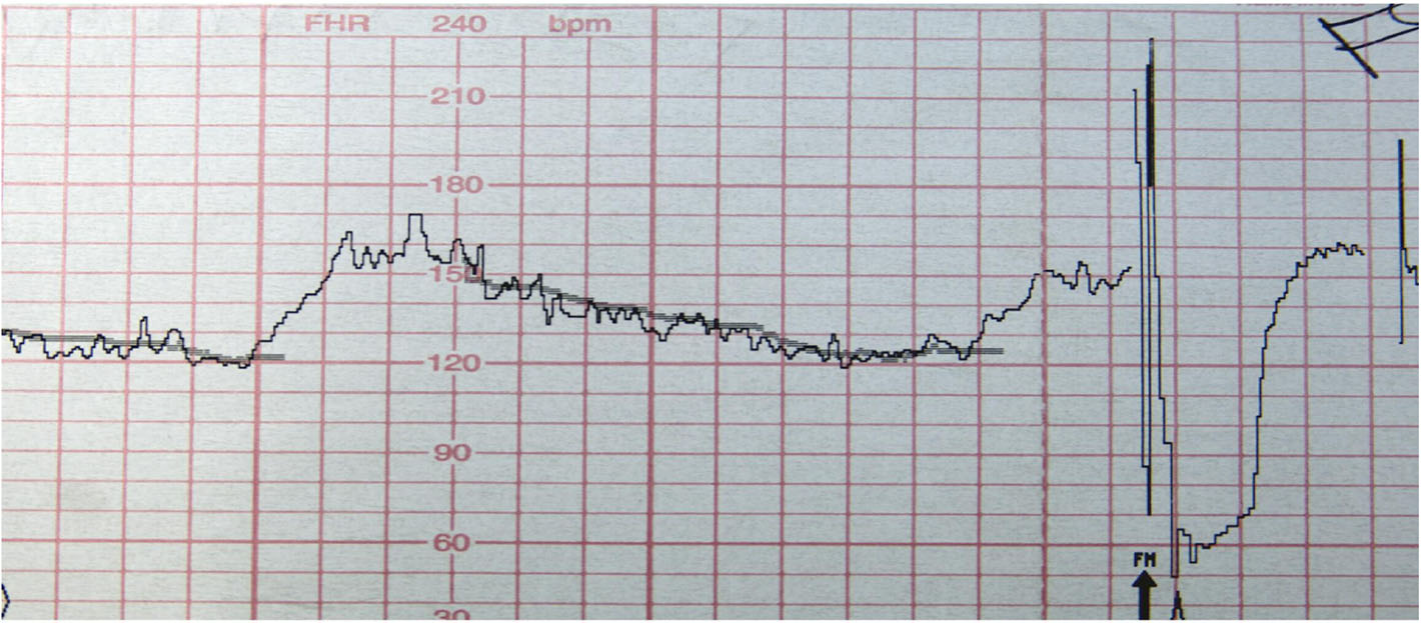
Illustrates coincidence between the maternal heart rate (faint line) and presumed fetal heart rate. *After application of a fetal scalp electrode, the true fetal heart rate becomes apparent. Reprinted from American Journal of Obstetrics and Gynecology,* https://www.sciencedirect.com/journal/american-journal-of-obstetrics-and-gynecology*, Vol 198, Issue 6, June 2008, Pages 717–24, Neilson DR, Freeman RK, Mangan S. Signal ambiguity resulting in unexpected outcome with external fetal heart rate monitoring.© 2008, with permission from Elsevier*

We acknowledge that not all CTG monitors in clinical use include signal ambiguity technologies and that, even when these technologies are employed and utilized correctly, it is possible that they still may miss MHRA or Type 2 FHRA. Therefore, we believe that in addition to signal ambiguity detection technologies, healthcare provider education regarding the clinical features of potential CTG artefact is essential, as this may be critical when these technologies are not available, malfunction, or in cases which are not detected by those technologies (for example, MHRA with doubling of the maternal heart rate). The clinical warning signs for possible MHRA are outlined in various publications and should be emphasized in clinician training: sudden sustained improvement of a tracing to a normal pattern, when it was previously of poor technical quality or showed an abnormal FHR; a “normal” FHR showing accelerations with every contraction (the maternal response to pain or pushing) (See Fig. 2); the apparent presence of marked accelerations and decelerations; sudden change to a new baseline and /or fluctuation in the baseline or fluctuation in the character of the tracing (variation in appearance of the variability sometimes accompanied by intermittent loss of contact); doubling/halving of the rate (this may also be a feature of FHRA) [1–4, 42, 43]. These features are especially significant when they begin after a position change, gap in monitoring, epidural insertion, and especially in the second stage of labour with onset of pushing. They are less common with application of a fetal scalp electrode which will often give the true fetal heart rate signal (see Fig. 3), but may, in some circumstances still occur, particularly after fetal death with recording of the maternal heart rate [44]. For Type 2 FHRA, the key feature which should raise concern is a narrow or no difference between the presumably different FHR signals in multiple gestation.

To facilitate knowledge translation, we have created a supplementary file which highlights the key points for clinicians (Additional file 4).

It is also useful to consider the larger research context. MHRA may manifest in two distinct ways with different clinical implications. One, the MHRA may mask a normal fetal heart rate, potentially leading to unnecessary intervention or, two, the MHRA may mask an abnormal fetal heart rate, potentially leading to false reassurance. Pinto et al. have demonstrated that the former is more common, manifesting most often as periodic switching to the MHR giving the false appearance of fetal decelerations [45]. Our cases seem more likely to involve the latter and this is probably because we are analyzing the most extreme cases, those involving fetal/neonatal death. Of note, simultaneous computerized analysis of MHR and FHR is an ongoing area of research which shows great promise as a future direction [46, 47]. By demonstrating improvements in detection of fetal acidemia by computer analysis of both maternal and fetal heart rates, research groups working in this area have provided additional evidence to support continuously monitoring the maternal heart rate along with the fetal heart rate [46, 47].

Our study has several limitations. Our a priori study design has certain weaknesses, in particular the lack of a pre-published protocol. Admittedly our study methodology would have been stronger if all 117 reports retrieved had been reviewed by all authors, but we believe that by having all authors review the positive cases (defined as those involving probable MHRA and/or a manufacturer’s recommendation regarding MHRA), our methodology is sufficiently strong for the case series reported here. Of note, our a posteriori presentation of results is rigorous and accessible: by making all our positive data points transparent and compact, an interested reader can review all our positive reports in a short amount of time and draw their own conclusions. Further our search strategy can be easily and independently validated by searching the FDA MAUDE database for product code “HGM” along with event type “death” which retrieves over 90% of the reports we found. Several factors mitigate against bias: our endpoint, death, is objective and our exposure, MHRA, has often been effectively assessed a priori and independent of our study by the authors of the FDA reports. That being said, without a full review of the CTG trace and history in each case, it is, admittedly, difficult to state definitively whether MHRA is truly present and a causal factor. The data is inherently messy and several of the reports are incomplete.

However, it is our opinion that our study design was uniquely suited to the problem we are trying to address and allowed us to thread the needle to an important clinical finding which continues to be under-recognized. Prospective clinical study of maternal heart rate artefact is likely to be hampered by the Hawthorne effect as relatively modest healthcare provider training and education in this topic is likely to greatly reduce the risks of adverse outcome. Further, a large number of cases would need to be studied. Neilson et al. reported that in approximately 10,000 deliveries, they observed 5 cases of unanticipated adverse perinatal outcome due to signal ambiguity [2]. Further, we anticipate that proposals for large healthcare-system based retrospective studies of fetal and neonatal deaths with an aim of determining if they were due to failure to detect MHRA and, potentially, failure of healthcare providers to follow manufacturer’s instructions for use, is likely to face significant barriers to approval due to medico-legal implications. Reviewing the FDA MAUDE database allowed us to sample a significant number of severe cases while circumventing the barriers listed above. Manufacturers have an obvious incentive to conclude that their CTG monitor was not at fault. However, we consider it significant that they so often concluded that the true problem was failure of the healthcare team to detect MHRA rather than failure to simply interpret the CTG tracing in general.

The FDA MAUDE database most likely suffers from important levels of under-reporting, potentially leading to an underestimation of the magnitude of the problem. The reasons to not report adverse outcomes are well known to clinicians: medico-legal concerns, potential for loss of reputation, medical license, and/or employment, lack of institutional support, lack of clear protocols for reporting, a belief that reporting will be futile, hierarchy and its associated problems, and the second victim syndrome [48] which can demoralize the healthcare provider in the aftermath of an adverse outcome. Further, there is an additional reason not to report to the FDA: uncertainty as to whether the CTG monitor or the healthcare provider was truly at fault. We would expect only those cases where the healthcare provider felt reasonably certain the CTG monitor was at fault to be reported to the FDA. However, if there is under-reporting, our conclusions are potentially more important than suggested by the number of cases reported here. In fact, they may only be the tip of the iceberg. Even with a rate considerably lower than 5 in 10,000 for unexpected adverse perinatal outcomes due to signal ambiguity, as estimated in 2008 by Nielson et al. [2], the clinical implications may be important due to the potentially preventable nature of the adverse events, the large number of births worldwide (estimated at more than 130 million) [49] and the low baseline risk of neonatal encephalopathy associated with intrapartum events (for example, estimated at 16 per 10,000 births with an associated death rate of 10% or 1.6 per 10,000 in for “level 1” countries with neonatal mortality rates less than 5 per 1000 live births) [50]. There is one caveat. While the number of cases reported to the FDA of fetal/neonatal death involving MHRA is most likely a gross underestimate of the number of such cases which occur, it also seems likely that the percentage of fetal/neonatal deaths which involve MHRA reported to the FDA MAUDE database (40% in our study) is an overestimate of the percentage in reality. The reason is that, while under-reporting leads to an underestimate of the magnitude of the problem of MHRA, knowledge gaps regarding MHRA on the part of healthcare providers and the subsequent confusion when there is an adverse outcome likely make such cases somewhat more likely to be reported.

The FDA MAUDE database website states that the reports should not be used to estimate rates of events [14]. That being said, our quantitative results are less important than the answer to the simple qualitative question: is there enough evidence here regarding the need for increased healthcare provider training in CTG artefact detection and management, in particular signal ambiguity detection technology, that the subject merits greater attention in CTG clinical practice guidelines? We believe that the answer to that question is yes. To quote a patient safety expert from another field, anesthesiologist David Gaba wrote that “no industry in which human lives depend on the skilled performance of responsible operators has waited for unequivocal proof of the benefits of simulation before embracing it.” [51] An FDA MAUDE database study such as ours can never provide unequivocal proof that increased healthcare provider awareness and training about MHRA will prevent adverse outcomes. However, ours is not a purely academic pursuit. With potentially preventable intrapartum injury and death to babies that might otherwise be born healthy in the balance, we believe that urgent dissemination of the results presented here is essential.

## Conclusion

MHRA is a persistent concern in the reports we have analyzed of fetal and neonatal death associated with cardiotocography, suggesting increased training and technological improvements are needed to mitigate this risk. Ideally, healthcare providers should be systematically trained through clinical simulation exercises focusing on verification of fetal life prior to CTG monitoring, signal ambiguity detection technologies, best response to coincidence alarms from those technologies, and best clinical acumen to detect MHRA. Finally, strong consideration should be given to updating cardiotocography guidelines to emphasize the risks of maternal heart rate artefact and the strategies to mitigate those risks, including signal ambiguity detection technologies and clinical vigilance.

## Supplementary information

**Additional file 1.** All 47 cases of fetal or neonatal death involving probable maternal heart rate artefact, reported to the US FDA from March 31, 2009 to March 31, 2019

**Additional file 2.** All 29 involving a recommendation from the manufacturer regarding maternal heart rate artefact and fetal or neonatal death, reported to the US FDA from March 31, 2009 to March 31, 2019

**Additional file 3.** 14 cases of intrapartum fetal death or neonatal death involving probable maternal heart rate artefact, reported to the US FDA from March 31, 2009 to March 31, 2019

**Additional file 4.** Clinical Summary: Diagnosis and Management of Cardiotocography Artefact

## Data Availability

All FDA reports referred to in this study are openly available to the public and, while they remain less than 10 years old, are accessible online from the US FDA MAUDE database (https://www.accessdata.fda.gov/scripts/cdrh/cfdocs/cfMAUDE/search.CFM). In addition, all the reports referred to in this study have been archived in the open access Figshare repository in the following dataset project: Kiely DJ, Oppenheimer LW, Dornan JC. Cardiotocography-associated fetal and neonatal deaths reported to the US FDA 2009–2019. Available at: https://figshare.com/projects/Cardiotocography-associated_fetal_and_neonatal_deaths_reported_to_the_US_FDA_2009-2019/67928 . Of note, the US FDA removes identifying information from reports in the MAUDE database before making the reports public.

## Abbreviations

CTG: Cardiotocography
EFM: Electronic fetal monitor
FDA MAUDE database: US Food and Drug Administration Manufacturer and User FacilityDevice Experience Database
FDA: United States Food and Drug Administration
FHRA: Fetal heart rate artefact
MHRA: Maternal heart rate artefact
