# Supplementary material for: Unrecognized maternal heart rate artefact in cases of perinatal mortality reported to the United States Food and Drug Administration from 2009 to 2019: a critical patient safety issue
Source: BMC Pregnancy Childbirth. 2019 Dec 16;19:501. doi: 10.1186/s12884-019-2660-5 (PMC6915916; doi:10.1186/s12884-019-2660-5)
Supplement: Supplementary file 1 — Additional file 1. All 47 cases of fetal or neonatal death involving probable maternal heart rate artefact, reported to the US FDA from March 31, 2009 to March 31, 2019 [file 12884_2019_2660_MOESM1_ESM.pdf]

Additional file 1: All 47 cases of fetal or neonatal death involving probable maternal heart rate artefact, reported to the US FDA from March 31, 2009 to March 31, 2019. (Note: all event types for reports listed here classified by the FDA as “death”)

NOTE:

Highlighted in yellow: sections which led to classification of the report as probably involving MHRA.

Highlighted in green: sections summarizing the event.

1) PHILIPS MEDICAL SYSTEMS AVALON FM30 FETAL MONITOR
Model Number M2703A
Event Date 02/03/2019

Event Description

The customer reported the death of a newborn after an emergency c-section and 19 minutes of attempted resuscitation. According to the customer (biomedical engineer) and caregivers, artifacts between the mother¿s pulse and the fetus' pulse rate were identified. But they think the device worked properly.

Manufacturer Narrative

A follow up report will be submitted once the investigation is complete. Serial number not provided at time of report.

MDR Report Key 8331468
Report Number 9610816-2019-00044

2) PHILIPS MEDICAL SYSTEMS AVALON FM50 FETAL MONITOR PERINATAL MONITORING SYSTEM
Model Number M2705A
Event Date 02/14/2018

Event Description

The customer reported a patient monitoring issue. The customer reported an incident with ¿undesirable results¿. The device was used for monitoring at the time of the alleged malfunction. An incident with ¿undesirable results¿ was reported. The customer did not provide any patient information, although the customer service manager tried to obtain further patient details.

Manufacturer Narrative

The actual monitor used in the incident was checked by a field service engineer (fse) onsite. The device successfully passed the performance verification and electrical safety tests. No issue with the device was found by the fse. The provided trace of the incident was evaluated by product support engineering (pse). The fetal heart rate (fhr) was derived from a cableless (cl) ultrasound (us) transducer. From a technical viewpoint, the derivation of the us signal was excellent, although at around 20:24, the us transducer did not record a signal, likely due to bad positioning of the transducer. At 20:28, the spo2 sensor was removed and the maternal pulse was derived by the cl toco mp transducer. This signal was lost intermittently. From 20:31 to 20:32, the trace shows movement artifacts. Between 20:32 to 20:39, there is no sufficient signal from the mother to allow coincidence detection. A reliable second pulse or heart rate source is required to perform the cross channel verification. From 20:39 onwards, the pulse was again derived by a spo2 sensor. The device issued coincidence alerts at 20:19, 20:22, 20:28, and 20:30 as intended by design. No technical malfunction was observed by pse. The trace was also clinically assessed by a philips physician and an external advisory midwife. They observed that in general the trace shows no accelerations and oscillations with limited undulations. In combination with the decelerations, this pattern presents a suspicious trace. The trace shows deceleration with consecutive loss of fhr baseline. At 20:35, the us transducer probably recorded the maternal pulse source instead of the fetal heart rate. In those cases, the maternal pulse will be shown in fhr trace on the print out. However, because between 20:30 and 20:39, the maternal pulse trace was being lost intermittently, there was not any second pulse or heart rate source at that time; therefore, a cross channel verification could not be performed. At 20:39, it appears that the us transducer switched back to the fetus. At 20:43, a fetal deceleration can be seen. The trace for the fetal signal ended at 20:45; possibly the transducer was removed from the mother¿s belly. The maternal pulse continued to be measured by the spo2 sensor until 21:00. The complete trace ended at approx. 20:57. A comprehensive trace analysis was not possible in this case as it requires additional data, such as therapy provided, patient history, etc. , which was not available in this case. The device worked as designed. No malfunction could be identified based on the provided information. The device remains at the customer site. The customer was informed via customer letter abut the outcome of the investigation. No further investigation or action is warranted.

MDR Report Key 7376854
Report Number 9610816-2018-00089

3) PHILIPS MEDICAL SYSTEMS AVALON FM50 FETAL MONITOR PERINATAL MONITORING SYSTEM
Model Number M2705A
Event Date 11/20/2017

Event Description

The customer called for application support to pull data from a monitor to see if alarms were visible and acknowledged. The customer reported that they had an adverse outcome where there was a concern with regard to heart rate coincidence between the fetal and the maternal heart rate. The device was used for monitoring at the time of the alleged malfunction. The customer stated that there was a delivery with an adverse outcome. No further details about the adverse event were made available by the customer.

Manufacturer Narrative

The issue was evaluated by the clinical specialist (cs) who checked whether the monitor's alarm configuration was set up as discussed with the customer during installation. The cs confirmed that all alarms were enabled correctly and the alarm pause mode was disabled. The cs stated that the trace of the particular adverse event showed question marks indicating the coincidence alarms (as intended when there is a coincidence between the measurements of fetal and maternal heart rate). Despite requested by the cs, the customer did not want to provide the trace for further evaluation by philips. Hence, no further investigation was possible. The cs confirmed that the alarm configuration was set correctly and that the trace of the particular adverse event   showed question marks indicating the coincidence alarms. The fse performed a functional check of the monitor at the customer site and confirmed that   the monitor worked as specified during testing. The product remains at the customer site. The device worked as intended and no malfunction of the device occurred. The alarm configuration was set correctly and the trace of the particular adverse event showed question marks indicating the coincidence alarms. No further investigation or action is warranted.

MDR Report Key 7063811
Report Number 9610816-2017-00373

4) PHILIPS MEDICAL SYSTEMS AVALON FM30 FETAL MONITOR
Model Number M2703A
Event Date 11/13/2017

Event Description

The customer reported that the (b)(6) monitor did not warn clearly enough of a coincidence and the printout was misleading or unclear. A newborn died when the device was used for monitoring during delivery.

Manufacturer Narrative

The customer stated that the warnings for a questionable fetal heart rate (fhr) were unclear to him, however, he suspected that the maternal heart rate (mhr) had been measured instead of the fhr. The customer complained that the monitor behavior is not clear and not sufficiently described in the instructions for use (ifu). According to the customer¿s problem description, the issue happened in the night from (b)(6) 2017. However, it was then determined based on the provided cardiotocograph (ctg) printout that the delivery took place in the night from (b)(6) 2017. The baby died on (b)(6) 2017. The service distributor was onsite to evaluate the reported issue and confirmed that no malfunction of the device was identified. Nevertheless, the biomedical engineer of the hospital sent the monitor to the

philips factory for an additional evaluation. The returned device was checked by product support engineering (pse) who confirmed that the monitor successfully passed the final test. The logs of the monitor showed coincidence alarms at the time of the reported incident which were silenced manually by a user. By silencing alarms, the user acknowledges all active alarms by switching off audible alarm indicators. Pse confirmed that the device showed no malfunction and worked as specified. The customer was instructed about the intended functionality which is considered as all that is warranted for this issue. The product remains at the customer site and is still in use in the labor and delivery ward. Additionally, the available information from this report does not support that this failure represents a systemic, design, or labeling problem. No further investigation or action is warranted.

MDR Report Key 7063061
Report Number 9610816-2017-00372

5) PHILIPS MEDICAL SYSTEMS AVALON FM20 FETAL MONITOR
Model Number M2702A
Event Date 08/13/2017

Event Description

The customer is questioning if the maternal heart rate (mhr) can be recorded as the fetal heart rate (fhr). There was a neonatal patient death reported 3 days after the delivery. The device was used for monitoring at the time of the alleged malfunction.

Manufacturer Narrative

During the birth on (b)(6), a drop of the fhr was seen at 14:40. The baby was born at 14:59 with an apgar score of 2, measured at 1 minute and 5 minutes after birth. The apgar score is a method to quickly summarize the health of newborn children. The baby passed away 3 days after birth due to hypoxic ischemic encephalopathy (hie). The customer provided the trace for the adverse event. The software version of the device is f. 01. 55. The trace it self was printed from a surveillance system, thus no information about the transducer and used monitor was available on the trace. The patient was monitored with a toco transducer and an ultrasound transducer. The maternal heart rate was not monitored, thus no coincidence notation between a maternal heart rate and a fetal heart rate was possible for instances when the ultrasound transducer detected the maternal pulse (e. G. From the aorta abdominalis) instead the fetal heart rate. The trace has been evaluated by a philips physician and an external midwife. They stated that the trace starts with showing signs of compromised fetal well-being through low variability. At 13:36 the fetal trace shows additional decelerations as another sign for clinical deterioration. At 14:06 the fetal trace changes again. The trace shows less decelerations and normal variability. The fhr did not react to contractions any more. This would be an unlikely, sudden clinical improvement of the fetus and is probably caused by the ultrasound switching to the prominent pulse source of the mother. The fetal heart movements are probably too weak or gone from that moment onwards. The device was tested by an engineer, no malfunction could be identified. The problem was solved by instructing the customer which is considered as all that is warranted for this issue. The product remains at the customer site and is used in the labor and delivery ward. Additionally, the available information from this report does not support that this failure represents a systemic, design, or labeling problem. No further investigation or action is warranted.

MDR Report Key 6951421
Report Number 9610816-2017-00333

6) PHILIPS MEDICAL SYSTEMS AVALON FM50 FETAL MONITOR PERINATAL MONITORING SYSTEM
Model Number M2705A
Event Date 05/26/2017

Event Description

The customer reported that a philips fm50 fetal monitor was in use during a critical incident that resulted in an fetal death. The device was used for monitoring at the time of the alleged malfunction.

Manufacturer Narrative

The customer stated there was an unexpected c-section due to the baby's failure to descend on (b)(6) 2017. The approximate duration of the birth was from 13:50 to 17:30. The customer did a c-section and thought everything was well, however, upon delivery the baby was deceased. The fm50 was removed from service by the hospital's clinical engineering department, and was returned to the factory together with the following devices: - device type : m2705a avalon fetal monitor fm50, serial number (sn): (b)(4) with fw revision: a. 06. 31. - device type: m2736a avalon us transducer, serial number: (b)(4), manufactured may 2016. - device type: m2734b avalon toco mp transducer, serial number: (b)(4), manufactured march 2017. The product support engineer (pse) conducted a performance test on the returned equipment. No trouble was found, the devices were fully operational and working as specified. The alarm review (in service mode) was checked on the monitor. A general test on the alarm functionality was performed, the alarming worked as specified. As the monitor was used after the incident, the alarms and stored data for the incident dated (b)(6) 2017, were not available anymore due to limited storage of traces and alarm history. The customer also provided the traces of the reported incident. The traces were reviewed by the product support engineer (pse) and an external senior midwife working closely with philips. During the evaluation of the traces, it was noted that the avalon toco mp transducer listed on the traces was not the same transducer that had been returned to the factory for evaluation (sn: (b)(4) on trace but sn: (b)(4) returned). Both pse and midwife observed that the cardiotocography is clinically conspicuous from the beginning. Restricted oscillation could be observed. The heart rate curve of the fetus is not fluctuating around the baseline when labor pain occurs. From 16:04 onwards, there is no safe recording of the child anymore. The mother was measured with the ultrasound transducer by mistake although the transducer actually should pick up the fetal heart rate.

Coincidence alarms were reported correctly and appear on the traces regularly and repetitively. There are no indications for a malfunction of the device in the recording. The customer additionally sent a second trace of another examination from the monitor. This trace also shows multiple coincidence alarms between the fetal heart rate and the maternal pulse. The trace shows that the device was working as specified during this examination as well. The avalon series fetal monitors utilize ultrasound technology to measure the fetal heart rate non-invasively. It is well documented in the avalon instructions for use (ifu), that phenomena/artifacts such as halving or doubling of the fetal heart rate, or switching between maternal and fetal heart rate can occur when using this method. The equipment was

sent back to the customer, 2 of 3 involved devices have been evaluated. No trouble could be found with the evaluated devices and traces. The problem was likely caused by insufficient knowledge of the functionality, and the customer was instructed accordingly. The products remain at the customer site.

MDR Report Key 6633726
Report Number 9610816-2017-00181

7) PHILIPS MEDICAL SYSTEMS AVALON FM30 FETAL MONITOR
Model Number M2703A
Event Date 11/29/2016

Event Description

In this case the customer reported a patient incident with a deceased fetus where it is now in question why the monitor showed a fetal heart rate but the fetus was deceased.

Manufacturer Narrative

The patient is a (b)(6) year-old (b)(6) female, primigravida, presenting with a pregnancy at (b)(6) with a recently diagnosed fetal demise. The patient presented to the emergency room on (b)(6) 2016 in the morning complaining of decreased fetal movement. On workup ultrasound was done revealing fetal demise. Labs were, otherwise, unremarkable on her workup. Ultrasound that was done in the radiology department revealed the fetal demise. No cardia motion. The estimated age of the fetus was (b)(6). The placenta was anterior with no previa. Cervical length was 3. 1 cm. Afi was 16. 2. On the patient¿s presentation to the emergency room, there was a fetal heart rate trace taken. It showed a fetal heart rate between 150s to 170s with some unusual hyper variability. No apparent decelerations looking at the fetal heart rate tracing retrospectively. The maternal heart rate was measured with a spo2 transducer and was between 80s and 90s bpm. The provided data (log files and strips) have been evaluated in the factory by a philips clinician and the product support engineer. The log files showed no fatal errors. Evaluation of the provided strips identified that the fetal heart rate presented in the trace are duplicated maternal heart rates. From a technical point of view this is not a malfunction. The algorithm measuring the fetal heart rate is designed to detect pulsations from the fetal heart. In case the fetal is already demised, the algorithm will take the next best pulsating signal which is the maternal aorta. Due to the demised fetus swinging in the anionic fluid within in the ultrasound beam, the algorithm easily might double or half the detected heart rate. This is one limitation of the auto correlation algorithm. The auto correlation algorithm can display a doubled fetal or maternal heart rate if the duration of diastole and systole are similar to each other, and if the heart rate is below 120 bpm. Doubling, usually brief, is accompanied by an abrupt switch of the trace to double the baseline value. In addition the maternal heart rate may simulate a normal fetal heart rate pattern (i. E. , it may mask a fhr deceleration or fetal demise). It was found that the fetal life was not confirmed before starting patient monitoring as it is strongly recommend by philips. Fetal movement profile (fmp) annotations on a fetal trace alone may not always indicate that the fetus is alive. The body of a deceased fetus can move and cause the monitor to annotate fetal body movements. This is what the customer has seen in this case on the strips. There had been movement annotations even when the fetus was already deceased. The customer has not confirmed the fetal life before starting with patient monitoring. In case the fetus is already deceased when doing fetal monitoring the device might misinterpret the maternal heart rate obtained from the aorta as fetal heart rate.

Due to the fact that deceased fetus is swimming in the anionic fluid in front of the aorta, these movements might be misinterpreted as fetal movement of a well being fetus. Thus this case falls into a usage of outside normal and expected. The results of the event investigation and device evaluation have been provided to the customer in a customer letter. There was no malfunction of the device. The device remains at the customer site. No part failed.

MDR Report Key 6171431
Report Number 9610816-2016-00314

8) PHILIPS MEDICAL SYSTEMS AVALON FM30 FETAL MONITOR
Model Number M2703A
Event Date 06/24/2016

Event Description

The customer reported the birth of a non-viable infant on (b)(6) 2016 at 21:18 after which the infant expired despite resuscitation efforts. The customer indicates that there was a discrepancy between the avalon fm30 cardiotocograph (ctg) data and the clinical outcome of the patient; the incident was not anticipated or expected based on the fetal monitor data.

Manufacturer Narrative

The provided trace has been evaluated by a philips rnd engineer and a philips clinician. Philips did not go on site to evaluate the device. Based on the evaluation, no product malfunction could be identified. Several warnings ¿check paper¿ and coincidence alarms have been printed on the trace indicating on one hand that the customer was not using philips paper and on the other hand that the device had detected a coincidence between the maternal pulse, obtained from the toco mp transducer and the fetal heart rate obtained from the ultrasound transducer. The available data supports that the device did not malfunction. The customer received a letter about the findings. Philips cannot determine if user error was a factor in the fetal demise, therefore any coincidences which the device alerted may have remained unrecognized. The device clearly indicated the detection of coincidence between the maternal pulse, obtained from the toco mp transducer and the fetal heart rate obtained from the ultrasound transducer.

MDR Report Key 5791986
Report Number 9610816-2016-00193

9) GE COROMETRICS FETAL MONITOR
Model Number 250CX SERIES MATERNAL/FETAL MONITOR
Event Date 11/11/2015

Event Description

Ge corometrics 250cx series maternal/fetal monitor produced inconsistent coincidence monitoring during high risk labor. The equipment failure may have been a contributory factor in the subsequent fetal demise. Used during labor until emergent c-section.

MDR Report Key 5413568
Report Number MW5060030

10) PHILIPS MEDICAL SYSTEMS AVALON FM50 FETAL MONITOR PERINATAL MONITORING SYSTEM
Model Number M2705A
Event Date 09/16/2015

Event Description

The customer submitted a pimr and stated there was a misidentification of maternal heart rate as fetal, and the fetus died.

Manufacturer Narrative

The philips clinical consultant and philips ob monitoring solution specialist had an onsite visit on (b)(4) 2015. Addressing the issues of reading mom vs baby on the strips and addressing protocol on monitoring both patients and distinguishing mom¿s hr by checking the radial pulse to confirm and compare to the hr on the strip. The philips clinical consultant and philips ob monitoring solution specialist onsite reviewed the monitors with the staff. No trouble was found. The philips clinical consultant also reinforced training with the avalon fm50 and transducer station. The philips clinical consultant also discussed using the spo2 to get a pulse if the maternal pulse is lost during a contraction or end stage labor. Over all there was no problem with the philips monitoring system identified. The customer was receptive, open with their discussion and feedback. The m2705a avalon fm50 fetal monitor s/n: (b)(4) was tested onsite by the philips clinical consultant and the customer. This evaluation has revealed no abnormalities and the device passed. Additional training about the ifu content was provided to resolve the use issues. The device remains at the customer site for use. No further investigation or action is warranted.

MDR Report Key 5155541
Report Number 9610816-2015-00246

11) PHILIPS MEDICAL SYSTEMS AVALON FM20 FETAL MONITOR
Model Number M2702A
Event Date 04/23/2015

Event Description

The customer stated that they were "monitoring with m2702a avalon fm20 fetal monitor s/n: (b)(4). Baby was born dead but the fetal monitor has recorded fetal heart rate (fhr) traces and fetal movements (fmp) although the fetus already was dead. User want to know how to identify it was mother's hr instead of fhr. There was fmp and contraction showed in the toco".

Manufacturer Narrative

Philips medical systems investigated the issue described above. On may 8, 2015, a philips field service engineer (fse) performed a functional test to verify the functionality of the avalon fm20 fetal monitor and determined that the device worked as specified. The provided trace recordings were reviewed by philips research & development department (r&d) and show: the heart rate baseline was around 120 bpm from 13:23 to 13:38 and from 14:16 to 16:08 with an increasing heart rate signal loss after

15:00. Movements were also recorded. From 18:01 to 18:48, only a toco transducer was applied (trace annotation ¿fhrx mode: no transducer¿). In addition a copy of the fetal monitor recording was provided, covering above mentioned time frames until 15:20. Based on the review of the provided traces and the additional information we received, it was determined that the philips device worked as specified. There is no indication that the clinicians verified fetal life before initiating fetal monitoring device labeling (instructions for use) instructs that ¿fetal monitoring technology available today is not always able to differentiate a fetal heart rate (fhr) signal source from a maternal heart rate (mhr) source in all situations ¿. This phenomenon is due to limitations of the technology and widely independent of the brand and model of the fetal monitor. In addition, an increased maternal pulse rate around and above 120 bpm is often seen with fetal demise. The maternal heart rate may be atypically high and therefore confused with that of a live fetus. Apparent fetal movement (fmp) may also be detected by the monitor but this may be a result of maternal movement causing the fetus to move within the amniotic fluid. Per philips (b)(4) and fse engineer, the m2702a avalon fm20 avalon fetal monitor with s/n: (b)(4) works as specified. No product malfunction. The m2702a avalon fm20 fetal monitor remains at the customer site. There is no indication of any malfunction of the m2702a avalon fm20 fetal monitor. The available information does not support that use of the device was contributory to the reported stillbirth. The cause of the stillbirth is unknown. Based on the review of the provided traces and the additional information we received, it was determined   that our device worked as specified. Device labeling (instructions for use) instructs that ¿fetal monitoring technology available today is not always able to differentiate a fetal heart rate (fhr) signal source from a maternal heart rate (mhr) source in all situations ¿. Users should confirm fetal life by independent means before starting to use the fetal monitor. Since a mhr trace can exhibit features that are very similar to those of a fhr trace, users should not rely solely on trace pattern features to identify a fetal source. Also, fetal movement profile (fmp) annotations on a fetal trace alone may not always indicate that the fetus is alive. The body of a deceased fetus can move and cause the monitor to annotate. Maternal signal sources may be picked up when using the ultrasound transducer and could lead to misidentification when the mhr is higher than normal (especially when > 100 bpm).

MDR Report Key 4756179
Report Number 9610816-2015-00093

12) PHILIPS MEDICAL SYSTEMS AVALON FM50 FETAL MONITOR PERINATAL MONITORING SYSTEM
Model Number M2705A
Event Date 03/30/2015

Event Description

The customer reported "using m2705a avalon fm50 fetal monitor with m2734b avalon toco mp transducer, were getting coincidence alarm due to maternal pulse the same or close to fetal heart rate hr, using m2736a avalon us transducer. The baby died at birth".

Manufacturer Narrative

The research and development department (r&d) reviewed the provided trace copy submitted to us and the provided response and found from a technical point of view, there is no indication of a product malfunction. Result: from a technical point of view the tracings look correct. There is no indication of equipment malfunction. Investigation summary: a philips field service employee retrieved the log and

configuration files from the device and provided these to r&d for analysis. R&d confirms that several device configuration settings were adapted by you to meet your needs. It was noted that the acoustical ccv inop warning was enabled to sound immediately with minimum volume of ¿4¿. The error log did not contain entries related to any device malfunctions. The following equipment was involved as documented on the header of the fetal trace (collectively, the ¿equipment¿): m2705a avalon fm50 fetal/maternal monitor, serial number: (b)(4), software revision j. 30. 59 (the ¿monitor¿). M2736a us transducer, serial number: (b)(4), software revision a.06.31 (the ¿us¿). M2734b toco mp transducer, serial number: (b)(4), software revision a. 06. 31 (the ¿toco mp¿) the trace recording, log and configuration files from the device were examined by the philips research and development department (¿r&d¿) and the results were as follows. R&d confirms that all of the equipment functioned as specified and that no malfunctions were identified. Fetal trace analysis: r&d has reviewed and analyzed the copy of the fetal trace in detail. The trace starts on (b)(6) 2015, 23:25, and ends on (b)(6) 2015, 00:52. Summary: the toco mp correctly documented the maternal pulse rate which went up above the fetal heart rate during pushing (2nd stage of labor). During contractions the ultrasound transducer picked up a maternal signal (¿maternal switching¿ artifact). This was confirmed by the spo2 sensor applied shortly after midnight. Later the ultrasound transducer continuously recorded a maternal pulse rate, as indicated repetitively by the question marks on the trace (cross-channel verification = ccv). Details: paper grid and page numbers are not visible on the trace copy provided (brightness/contrast too high). A separate copy of the trace header showed equipment information (serial number, software revision) as documented above, printed at 21:21. International paper scaling with paper speed 3 cm/min has been used. Page 1 of the trace copy starting around 23:25: the records show that the us and toco mp transducers were plugged in, but only the us trace is visible on the trace copy. Movement bars are printed (fetal movement profile = fmp). In the 2nd stage of labour these movements mostly are of maternal origin. Page 2: the toco and mp recordings are starting. Between contractions the maternal pulse is 20 to 30 bpm below the fetal heart rate. During uterine contractions (pushing) the maternal pulse goes up above the fetal heart rate and the ultrasound temporarily switches to the maternal rate, indicated by the toco mp trace. Pages 3 to 5 starting at around 23:39: uterine contractions with pushing continue with strong maternal pulse rate accelerations. A uterine contraction may result in moving the fetal heart temporarily out of the us beam, and the signal from a maternal vessel can be picked up during this time. The cross-channel verification (which is indicated by ccv question marks on screen and on top of the recording) correctly indicated that the us and toco mp picked up a signal from the same source, i. E. Maternal. Between contractions the ultrasound returns to the fetal signal.

Page 5: after 23:53 the ultrasound almost continuously records a maternal signal. Ccv warning is given repeatedly. Page 6: at 00:02 the records indicate that a spo2 sensor has also been applied to the patient which automatically replaced the maternal pulse trace from toco mp. The trace patterns shown by the spo2 sensor are consistent with the trace patterns previously recorded by toco mp. This confirms that toco mp has correctly picked up the maternal and not the fetal pulse rate. Note: toco mp can pick up a fetal pulse rate only if a fetal artery is extremely close to the optical sensors of the transducer.

Pages 7 to 13: the fetal monitor correctly gave ccv warning as documented on the paper. During a heart rate coincidence condition the affected heart rates are marked on the fetal monitor screen with a question mark. In addition an acoustical inop is given (software revision j. 30). Pages 9, 11, 13: ccv warning is given although only the ultrasound trace is printed: spo2 had signal loss (not applied to the

patient?) and the maternal pulse trace (toco mp) on the recorder had been manually switched off. The ccv feature continues to work even if the mp trace recording is disabled. Accordingly, the tests and analysis performed by r&d confirm that the device worked as specified. There is nothing in the records to indicate any device or equipment malfunction. The baby died at birth. The customer would like to know if m2705a avalon fm50 fetal monitor s/n: (b)(4) worked properly. The m2705a avalon fm50 fetal monitor s/n: (b)(4) was used at the time of the stillbirth for monitoring. After fetal trace analysis per r&d, no indication of any malfunction was found. The m2705a avalon fm50 fetal monitor s/n: (b)(4) remain at the customer site. There is no indication of any m2705a avalon fm50 fetal monitor malfunction.

The device was not contributory to the reported stillbirth. The cause of the baby's death is unknown. No further investigation or action is warranted. (b)(4).

MDR Report Key 4710453
Report Number 9610816-2015-00081

13)PHILIPS MEDICAL SYSTEMS AVALON FM50 FETAL MONITOR
Model Number M2705A
Event Date 10/14/2014

Event Description

The customer reported that a female who has had one pregnancy and no live births presented in active labor ((b)(6)) to labor and delivery with contractions. During the second stage of labor, an emergent c-section was required due to fetal bradycardia that was not apparent on the electronic fetal monitor until an internal fetal scalp electrode was placed. At the time the bradycardia was discovered, the c-section was performed. The baby had a triple nuchal cord, born with no detectible heart rate and neonatal resuscitation program was initiated. The pt was transported to the nicu in critical condition.

The infant was resuscitated post-partum, and transferred to (b)(6) where they determined the infant had no brain activity thus transferred back. This was a post-term infant with (b)(6) complete weeks of gestation. The baby was delivered asystolic, and required full resuscitation including intubation, positive pressure ventilation (ppv), chest compression, multiple epinephrine doses and central line placement. Her apgars where 0, 0, and 0 at 1, 5 and 10 minutes. Heart rate was obtained approximately 13 minutes into resuscitation. Severe hypoxic ischemic encephalopathy; the family with drew life support and infant expired two days later.

Manufacturer Narrative

(b)(4). A follow up report will be submitted after philips obtains more information concerning this event.

MDR Report Key 4351506
Report Number 9610816-2014-00316

14) PHILIPS MEDICAL SYSTEMS AVALON FM30 FETAL MONITOR
Model Number M2703A
Event Date 07/02/2014

Event Description

The customer stated after a c-section the a baby was born dead/stillborn and the customer states that the m2703a avalon fm30 fetal monitor s/n: (b)(4) did pick up a fetal heart rate.

Manufacturer Narrative

(b)(4). A follow up report will be submitted once the investigation is complete.

MDR Report Key 3952283
Report Number 9610816-2014-00190

15) PHILIPS MEDICAL SYSTEMS AVALON FM50 FETAL MONITOR
Model Number M2705A
Event Date 01/14/2014

Event Description

The customer reported that the fetal monitor traced the mother's heart treat as the fetus. The baby is decreased.

Manufacturer Narrative

(b)(4). A follow up report will be submitted after philips obtains more information concerning this event.

MDR Report Key 3640710
Report Number 9610816-2014-00029

16) PHILIPS MEDICAL SYSTEMS AVALON FM50 FETAL MONITOR
Model Number M2705A
Event Date 07/31/2013

Event Description

The customer reported a fetal death while monitoring with an avalon fm50 fetal monitor. The customer requested that an audible inop for the coincidence detection alerts be introduced.

Manufacturer Narrative

(b)(4). A follow-up report will be submitted after philips obtains more information concerning this event.

MDR Report Key 3299684
Report Number 9610816-2013-00173

17) PHILIPS MEDICAL SYSTEMS AVALON FM50 FETAL MONITOR
Model Number M2705A
Event Date 08/02/2013

Event Description

The customer reported that the avalon fm50 fetal monitor was wrongly tracing a heart rate, and recorded a heart rate last night on a baby that had been born deceased.

Manufacturer Narrative

(b)(4). A follow up report will be submitted after philips obtains more information concerning this event.

MDR Report Key 3504303
Report Number 9610816-2013-00180

18) PHILIPS MEDICAL SYSTEMS AVALON FM20 FETAL MONITOR
Model Number M2702A
Event Date 06/10/2013

Event Description

The customer reported that the avalon fm20 fetal monitor was wrongly tracing a fetus that was already deceased/stillborn.

Manufacturer Narrative

(b)(4). A follow-up report will be submitted after philips obtains more info concerning this event.

MDR Report Key 3213220
Report Number 9610816-2013-00143

19) HUNTLEIGH HEALTHCARE LTD. DIAGNOSTICS SONICAID FM800 RANGE OF FETAL MONITORS
Model Number FM830ENCORE

Event Description

Clinicians have had difficulty distinguishing between the fhr and mhr. The customer did an operation and a fetus was still born yet the fhr was showing ok.

Manufacturer Narrative

(b)(4) is submitting the report on behalf huntleigh (b)(4). ((b)(4)) for more details.

MDR Report Key 3030183
Report Number 1000589001-2013-00002

20) HUNTLEIGH HEALTHCARE LTD. DIAGNOSTICS SONICAID FM800 RANGE OF FETAL MONITOR
Model Number FM830
Event Date 12/28/2011

Event Description

A communication was received from the adverse incident centre of the (b)(6), stating that an incident had been reported by (b)(6) hospital with regard to an fm830 fetal monitor. The reported fault with the monitor was "ctg displaying trace with fetal heart rate of 150bpm, but baby was dead. ".

Manufacturer Narrative

This report is being filed under exemption (b)(4) on behalf of the manufacturer huntleigh healthcare ltd. (registration#1000589001). The unit is being returned from (b)(6) hospital to the service department at (b)(6) for investigation. Additional information will be provided following the conclusion of the manufacturer's investigation.

MDR Report Key 2444284
Report Number 1000589001-2012-00001

21) PHILIPS MEDICAL SYSTEMS AVALON FM20 FETAL MONITOR
Model Number M2702A

Event Description

The customer reported receiving a heart rate on a fetus that was deceased.

Manufacturer Narrative

(b)(4). The customer reported receiving a heart rate on a fetus that was deceased. Philips is in the process of obtaining additional info concerning this event and the complaint is still under investigation. A final report will be submitted once the investigation is completed.

MDR Report Key 2168512
Report Number 9610816-2011-00387

22) PHILIPS MEDICAL SYSTEMS AVALON FM30 FETAL MONITOR
Model Number M2703A
Event Date 06/01/2011

Event Description

The customer reported that while monitoring with a philips avalon fm30 fetal monitor, a fetus was stillborn.

Manufacturer Narrative

(b)(4). The customer reported that while monitoring with a philips avalon fm30 fetal monitor, a fetus was stillborn. According to the doctor, the system functionality is not according to specification as there were heart sounds although the fetus was stillborn. The available information gives no indication that these users verified fetal life before initiating monitoring (as specified in device labeling). Philips is in the process of obtaining additional info regarding this incident and the complaint is still under investigation. A final report will be submitted once the investigation is completed.

MDR Report Key 2129896
Report Number 9610816-2011-00324

23) PHILIPS MEDICAL SYSTEMS AVALON FM30 FETAL MONITOR
Model Number M2703A
Event Date 03/14/2011

Event Description

The customer reported that during the use of an avalon fm30 fetal monitor, there was a stillbirth.

Manufacturer Narrative

(b)(4). The customer reported that during the use of an avalon fm30 fetal monitor, there was a stillbirth. The mother had a heart frequency of 60-80 bpm and the fetal had a heart frequency of 120 to 140 bpm. The initial info indicated that the baby had been dead for several days before monitoring began. There is no indication that the clinicians verified fetal life before starting monitoring (as specified in the device labeling, instructions for use). The avalon fm30 fetal monitor is still in use at the site. This is being reported only because use of the device was coincident with the stillbirth. Philips is in the process of obtaining additional info regarding this incident and the complaint is still under investigation. A final report will be submitted once the investigation is completed.

MDR Report Key 2039045
Report Number 9610816-2011-00163

24) PHILIPS MEDICAL SYSTEMS AVALON FM30 FETAL MONITOR
Model Number M2703A

Event Description

The customer reported that a baby died after being monitored by a philips device.

Manufacturer Narrative

(b)(4). The customer reported that a baby death occurred after being monitored by a philips device. This is being reported only because a philips device was in use on a baby who died. Based on the current, available information, the maternal heart rate (mhr) increased and coincided with the fetal heart rate (fhr), however, the fetal monitor showed/printed question marks. According to the statement from the head physician, the question marks were either ignored or not correctly interpreted due to human error. The baby suffocated during the birth. There is no indication of any malfunction of the (b)(6) avalon fm30. Philips is in the process of obtaining additional information regarding this incident and the complaint is still under investigation. A final report will be submitted once the investigation is completed.

MDR Report Key 2012314
Report Number 9610816-2011-00116

25) PHILIPS MEDICAL SYSTEMS AVALON FM30 FETAL MONITOR
Model Number M2703A

Event Description

The customer reported that an infant death occurred while being monitored on a philips device.

Manufacturer Narrative

(b)(4). The customer reported that an infant death occurred while being monitored on a philips device. Due to this request, it is considered that it was unclear for the customer how a fetal heart rate (fhr)

could be measured on a fetus which is death for 2 days. The available information supports that the infant death occurred before the hospital began monitoring using the philips fetal monitor. The device documentation (instructions for use) stresses to confirm fetal life by independent means prior to initiating monitoring. Per the philips response center engineer (rce), there is no indication of fetal life. Per a philips registered nurse (rn), a review of the provided trace showed that no continuous measurement was used to gather the maternal pulse. Therefore, it was not possible for the device to compare the maternal heart rate (mhr) with the fhr to announce the user of a potential coincidence. Note that the device labeling instructs users to use coincidence detection (ccf) to assure that the measured heart rate is not the maternal heart rate. In addition, please note that the customer has provided 2 trace snippets from 2 different days ((b)(6) 2011) and two different devices for review. Philips will report this incident separately for both involved devices. Philips is in the process of obtaining additional information regarding this incident and the complaint is still under investigation. A final report will be submitted once the investigation is completed.

MDR Report Key 1998690
Report Number 9610816-2011-00090

26) PHILIPS MEDICAL SYSTEMS AVALON FM30 FETAL MONITOR
Model Number M2703A

Event Description

The customer made an allegation of a unappropriated c-section and fetal death.

Manufacturer Narrative

The customer made an allegation of a unappropriated c-section and fetal death. Philips has not verified that there was a fetal death or what was the condition of the fetus when monitoring began. Philips is in the process of obtaining additional info regarding this incident and the complaint is still under investigation. A final report will be submitted once the investigation is completed.

MDR Report Key 1957267
Report Number 9610816-2010-00865

27) PHILIPS MEDICAL SYSTEMS AVALON FM20 FETAL MONITOR
Model Number M2702A

Event Description

The customer reported that they detected a normal fetal heart rate (fhr) whereas the baby had previously died in utero.

Manufacturer Narrative

(b)(6): the customer reported that they detected a normal fetal heart rate (fhr) whereas the baby had previously died in utero. The report is fully consistent with failure to verify fetal life before beginning to monitor and with placement of the ultrasound transducer so that the mother was monitored. Per the instructions for use (ifu), fetal life should be verified before monitoring and the ccv (cross channel

verification) functions should be used to alert clinicians if maternal heartrate (hr) is detected instead of fetal heartrate (fhr). Philips is in the process of obtaining additional info regarding this incident and the complaint is still under investigation. A final report will be submitted once the investigation is completed.

MDR Report Key 1809185
Report Number 9610816-2010-00325

28) PHILLIPS MEDICAL PHILLIPS AVALON FETAL MONITOR
Model Number AVALON FETAL
Event Date 05/30/2010

Event Description

Still birth of a full term infant. Phillips medical fetal monitor indicated the infant had a heartbeat until the exact time of birth. At birth there was no heartbeat or respiratory effort. There was no response from infant despite a prolonged resuscitation process. There is now a question as to the accuracy of the fetal monitoring equipment. Was it recording the maternal heart rate after the infant unknowingly died in utero? this was an uncomplicated (b) (6) pregnancy. Autopsy was normal except for meconium present in the lungs as well as histiocytes that had ingested meconium. Also note that phillips had released an urgent medical device recall related to this equipment. (b) (4).

MDR Report Key 1752476
Report Number MW5016627

29) PHILIPS MEDICAL SYSTEMS FETAL MONITOR SERIES 50 IP
Model Number M1353A
Event Date 06/05/2010

Event Description

The customer reported that a death occurred while a pt was being monitored by a philips fetal monitor.

Manufacturer Narrative

The customer reported that a death occurred while a pt was being monitored by a philips fetal monitor. The customer reported that the fetal monitor had given the baby's heart rate between 140 and 160bpm and then a range between 80bpm and 175bpm. Even when the baby was taken out of his mother, the monitor continued to show a rate of 140bpm whereas the baby was delivered stillborn. The available info is fully consistent with the clinicians monitoring the mother instead of the fetus. The available info is not sufficient to support that the use of the monitor was a factor in the stillbirth. Philips is in the process of obtaining additional info regarding this incident and the complaint is still under investigation. A final report will be submitted once the investigation is completed. (b) (4).

MDR Report Key 1729585
Report Number 9610816-2010-00195

30) PHILIPS MEDICAL SYSTEMS FETAL MONITOR

Model Number M1351A

Event Description

The customer reported that when they connected the mother to the monitor, they were receiving traces even though they were aware that the baby had died at least 24 hours before the monitoring began.

Manufacturer Narrative

The customer reported that when thy connected the mother to the monitor; they were receiving traces even though they were aware that the baby had previously died. The initial info is most consistent with monitoring the mother instead of the baby. Product labeling (instructions for use) adequately describes verification of fetal viability before monitoring and differentiation between the mother's heart rate (hr) and the baby's hr. Philips is in the process of obtaining add'l info regarding this incident, and the complaint is still under investigation. A final report will be submitted once the investigation is completed. (b) (4).

MDR Report Key 1697304
Report Number 9610816-2010-00163

31) PHILIPS MEDICAL SYSTEMS AVALON FM30 FETAL MONITOR

Model Number M2703A

Event Description

The customer reported that a ctg machine was recording a trace via a fetal scalp electrode on a fetus that had expired.

Manufacturer Narrative

The customer reported that a ctg machine was recording a trace via a fetal scalp electrode on a fetus that had expired. The available information supports that this was a user misunderstanding and not a malfunction. Philips is in the process of obtaining additional information concerning this event and the complaint is still under investigation. A final report will be submitted once the investigation is completed. (b) (4).

MDR Report Key 1666075
Report Number 9610816-2010-00112

32) PHILIPS MEDICAL SYSTEMS AVALON FM30 FETAL MONITOR

Event Date 02/09/2010

Event Description

The customer reported that while monitoring a pt with periodic uterus contractions, using a series 50 monitor, the fetus was found in 3rd stage of maceration.

Manufacturer Narrative

The customer reported that while monitoring a pt with periodic uterus contractions, using a series 50 monitor, the fetus was found in the 3rd stage of maceration. The report of the fetus being macerated supports that the death was well before the initiation of monitoring and was unrelated to the monitoring. Based on the available info, the physician documented that the mistake made was that they did not measure the mother's heart rate (mhr) and there is no indication of a product malfunction. Philips is in the process of obtaining additional info regarding this incident, and the complaint is still under investigation. A final report will be submitted once the investigation is completed. (b) (4).

MDR Report Key 1634970
Report Number 9610816-2010-00072

33) PHILIPS MEDICAL SYSTEMS AVALON FM50 FETAL MONITOR
Model Number M2705A
Event Date 01/07/2010

Event Description

On february 12, 2010, philips received a medwatch report stating that the baby died one day after the delivery.

Manufacturer Narrative

On february 12, 2010, philips received a medwatch stating that the baby died one day after the delivery. The customer made it clear in the medwatch that there have been difficulties distinguishing between the baby's and the maternal hr. And therefore, a c-section was performed. The customer made it clear that the clinicians were aware of the issue and made no allegation or indication that the device contributed to the death. In addition, there is no allegation of a product malfunction. Philips is in the process of obtaining additional info regarding this event and the complaint is still under investigation. A final report will be submitted once the investigation is completed. (b) (4).

MDR Report Key 1622600
Report Number 9610816-2010-00042

34) PHILIPS MEDICAL SYSTEMS AVALON FM30 FETAL MONITOR
Model Number M2703A

Event Description

The customer reported an incident where the ctg tracing for the fetal heart rate (fhr) was picking up the maternal heart rate (mhr) during labor and showing false positives. The hosp may have detected that the baby died in utero.

Manufacturer Narrative

The customer reported an incident where the ctg tracing for the fetal heart rate (fhr) was picking up the maternal heart rate (mhr) during labor after the hospital had detected that the baby died in utero. The clinicians verified that the fetus had no fhr by performing an ultrasound. The available info indicates that, during the verification of fetal viability that is normal clinical practice for fetal monitoring, the clinicians applied the internal fetal scalp electrode to the mother instead of the baby and derived the mhr instead of the fhr. In addition, the fetal monitoring by ultrasound also only could detect the mhr.

The product labeling (instructions for use) warns clinicians to verify fetal life before beginning monitoring. There is no indication of any malfunction of the monitoring equipment or labeling (ifu). Philips is in the process of obtaining add'l info regarding this event and the complaint is still under investigation. A final report will be submitted once the investigation is completed. (b) (4).

MDR Report Key 1618342
Report Number 9610816-2010-00041

35) PHILIPS HEALTHCARE, INC. PHILIPS AVALON FM50 FETAL MONITOR
Model Number FM 20
Event Date 11/30/2009

Event Description

A pt presenting to labor and delivery in labor. At 41 week gestation and history of prior planned c-section for twins. Patient placed on fm20 philips avalon. Monitor was erratic with tracings and at one point, it was difficult to distinguish mom and baby's heart rate on the tracing, but the digital readout was 30 beats apart. Ultrasound showed no heart beat. Emergency c-section was performed and the infant had an apgar of 0,0. The infant was resuscitated and transferred to facility with higher level of care in nicu. The baby did not survive, dying the next day.

MDR Report Key 1579412
Report Number 1579412

36) PHILIPS MEDICAL SYSTEMS AVALON FM20 FETAL MONITOR
Model Number M2702A
Event Date 08/07/2009

Event Description

The customer reported that a pregnant pt was admitted and although had a normal fetal hr, a caesarian section found the baby had macerated at least 48 hours prior to the surgery.

Manufacturer Narrative

This adverse event is being considered as reportable since it is a death where an allegation has been made that the device was a factor in the death. It is confirmed that the baby was dead for more than 48 hours. It is not confirmed for how long the monitoring was ongoing before it was decided to do the c-section (--more or less than 48 hours?). Therefore, we cannot confirm from the available info that the use of this device was not a factor in the death. This event was reported to the philips on 10/28/2009. We have not been able to determine if any philips representatives were aware of this event before 10/28/2009. The customer did not measure the mother's heart rate with a continuous measurement (spo2 and mecg) so the cross channel verification (ccv) algorithm could not generate warnings if the clinicians were monitoring the mother instead of the baby. The labeling is clear that the user should confirm the fetal life with an independent means before using the fetal monitor. Doing this would have detected that the baby might have been already dead. We will consider that the use outside that described in the labeling--neither confirming life before monitoring nor using the ccv functionality--delayed the clinician knowing that the baby was dead. The available info does not allow us to determine if knowing the status of the baby could have impacted the outcome. Philips is in the

process of investigating this event and a final report will be submitted once the investigation has been completed.

MDR Report Key 1545883
Report Number 9610816-2009-00205

37) PHILIPS MEDICAL SYSTEMS AVALON FM20 FETAL MONITOR
Model Number M2702A
Event Date 10/19/2009

Event Description

The customer reported that the ctg was giving a fetal rate reading of 160 bpm, but yet, the baby was stillborn after an emergency c-section was performed.

Manufacturer Narrative

The customer did not measure the mother's heart rate with a continuous measurement (spo2 or mecg), so the cross channel verification (ccv) algorithm could not generate warnings. The labeling (ifu) is clear that the user should confirm the fetal live with an independent means before using the fetal monitor. The device was tested afterwards and no malfunction was found. Philips has not determined if failing to use any ccv or verification of fetal life prevented some therapy that might have changed the outcome. Philips is in the process of obtaining additional info regarding this event, and the complaint is still under investigation. A final report will be submitted once the investigation is completed.

MDR Report Key 1545370
Report Number 9610816-2009-00206

38) PHILIPS MEDICAL SYSTEMS AVALON FM20 FETAL MONITOR
Model Number M2702A

Event Description

The customer reported suspicious trace at the central monitoring/nurse station. The customer reported us signal had no typical doublebeat.

Manufacturer Narrative

The clinicians chose emergency c-section because of the suspicious trace. The baby was already deceased. Philips is in the process of obtaining additional information concerning this event and the complaint is still under investigation. A final report will be submitted once the investigation is completed.

MDR Report Key 1409842
Report Number 9610816-2009-00070

39) PHILIPS MEDICAL SYSTEMS ULTRASOUND TRANSDUCER FOR USE WITH FM20/FM30 AVALON
Model Number M2736A
Event Date 10/12/2018

Event Description

It was reported that there was a "wrong ecg trace issue. The users reported that the ultrasound transducer didn't give the right ecg trace, the ecg trace was shown for a healthy baby's heartbeat, but the baby was born dead".

Manufacturer Narrative

A follow-up report will be submitted upon completion of the investigation. The device was in use on a patient at the time of the reported event. No further details regarding the baby had been made available at the time of the reporting decision. Philips is in the process of obtaining additional information regarding the reported event.

MDR Report Key 8032345
Report Number 9610816-2018-00303

40) PHILIPS MEDICAL SYSTEMS ULTRASOUND TRANSDUCER FOR USE WITH FM20/FM30 AVALON
Model Number M2736A
Event Date 01/24/2012

Event Description

The customer sent an email query regarding a field action in 2009 for philips avalon fetal monitors. This was in relation to a case regarding a baby who died in (b)(6) hospital in 2012. A newborn died when the device was used for monitoring during delivery at (b)(6) 2012. The incident took place in the hospital delivery room.

Manufacturer Narrative

A baby died on (b)(6) 2012. The incident was not reported to philips at that time. The investigation determined that the customer requested assistance to clarify questions related to the field safety notice from 2009 concerning the ultrasound (us) transducer signal (i. E. Device detecting the fetal heart rate (fhr)). This complaint is registered on the us transducer as the doppler echoes are processed by the mainboard within the ultrasound transducer by an auto-correlation algorithm to determine fetal heart rate (fhr). The signal processing of the fhr is done by the firmware (software) on the transducer mainboard. The fhr is only reported on the monitor¿s numeric display and on the recorded trace. The full traces of the incident have been provided by the (b)(4) to philips for an evaluation by product support engineering (pse) and a clinician. Pse stated that the traces show that the customer used multiple devices during that patient monitoring episode. A philips physician and an external midwife assessed the traces and concluded the following: the trace shows fetal distress starting at 06:32 am. From that moment onwards, the fhr trace rarely shows signals from the fetus, and is instead almost exclusively showing a maternal signal. This phenomenon is well-known and inherent to the fetal monitor¿s ultrasound technology. Therefore, the fetal monitor is designed to compare a known maternal signal (e. G. Pulse measured by an spo2 finger sensor) with the ultrasound signal. This coincidence analysis is continuously done by the monitor and alerts the user in case of a coincidence. Here, the coincidence analysis was only intermittently possible because the maternal spo2 probe was

not used continuously after epidural anesthesia was started. However, during those periods when the spo2 sensor was applied to the mother, the fetal monitor issued multiple coincidence alerts consistent with its design and labeling. The strips provided to philips show no indication of device malfunction. The investigation reveals that there is no relation between the death on (b)(6) 2012 and the field actions from 2009. The traces provided to philips show no indication of a device malfunction. The products remain at the customer site. The provided information shows that all avalon devices involved in the incident had the latest firmware on (b)(6) 2012. This complaint does not represent a product/part failure. No further investigation or action is warranted.

MDR Report Key 7233465
Report Number 9610816-2018-00035

41) WIPRO GE HEALTHCARE PRIVATE LTD COROMETRICS MONITOR PERINATAL MONITORING SYSTEM
Model Number 259 CX-C
Event Date 08/05/2012 Event Description

Ge healthcare has received notification of a death of a fetus.

Manufacturer Narrative

The legal complaint that general electric company received alleges the following: "the corometrics monitor made it appear to the healthcare providers that the fetal heart rate was being monitored throughout labor and was normal. " "however, at some point hours before delivery, his fetal heart rate became distressed. Instead of picking up this distress, the corometrics fetal heart monitor made a smooth transition to the maternal heart rate, confusing the healthcare providers into believing the baby's heart was fine. " ge healthcare's investigation is ongoing. A follow up report will be submitted once the investigation has been completed.

Manufacturer Narrative

No information has been provided to ge healthcare by the hospital on the status of the unit. At this time, it is not known if the unit was taken out of service   or if it continues to be used with patients. No further details about the alleged device or the event have been provided to ge healthcare. There are no service records for the device in the ge healthcare database. Therefore, without sufficient information about the event, or the evaluation of the alleged device, it is not possible to determine the root cause of the alleged issue.

Manufacturer Narrative

The following information was obtained by ge healthcare through the legal proceedings related to this case. Multiple clinical signs presented that indicated fetal distress during the monitoring session to which the ob team did not take appropriate actions: low fetal heart rate variability- a healthy fetus has high heart rate variability. Maternal heart rate was very close to fetal heart rate; the strip chart indicated the maternal heart rate and fetal heart rate actually overlapped 5 times during the monitoring session, as shown by the hbc indication- a healthy fetal heart rate is typically higher than the maternal heart rate. Fetal heart rate accelerated during contractions- a healthy fetus would have heart rate decelerations during maternal contractions. Multiple maternal parameters to indicate the mother was

at high risk for a complicated birthing process, i. E. She was overweight, tachycardic, and feverish. The ob team attempted to use a fetal scalp electrode (fse) to obtain a fetal heart rate directly on four occasions with two different electrodes. They observed no good signal from the fse attempts and concluded the electrodes were defective, instead of concluding there was a problem with the fetus. One of the obstetricians communicated they do not look at the monitor strip chart at all. Another member of the ob team communicated they didn't know about overlapping heartrates and, therefore, did not understand the indications. The obstetricians communicated they did not read the monitor user's manual and did not understand heartbeat coincidence. The obstetricians applied, removed, and then applied spo2 again. When spo2 was applied and indicated a maternal heart rate that was overlapping with the fetal heart rate, appropriate actions were not taken. The obstetricians did not consider the two heart beats were both from the mother and that the fetal heart beat was not being detected. Ge healthcare provided training on the device which was attended by hospital staff several months prior to the incident. The hospital is responsible for ensuring their clinicians are properly trained on the device through personnel turnover cycles, etc. The ge training was provided one time only and was not purchased again for new clinicians that did not receive the original training. It was concluded that the root cause was user error interpreting the clinical situation of the mother and the fetus.

MDR Report Key 3369763
Report Number 9617277-2013-00001

42) HUNTLEIGH HEALTHCARE LTD. DIAGNOSTICS SONICAID
Model Number FM830ENCORE
Event Date 08/29/2014

Event Description

The monitor was recording traces for an intra uterine fetal death.

Manufacturer Narrative

Arjohuntleigh, inc is submitting the report on behalf of huntleigh healthcare ltd. Exemption no. (b)(4). Having reviewed the limited information provided (pdf of trace provided dated (b)(6) 2014), the following points summarise the investigation: this would appear to have been a known high risk pregnancy, as indicated by the "g12p0" or more likely "g2p0" annotation (difficult to read) whether it's g12 or g2 it indicates that at least one, and possibly as many as 11, previous pregnancy(s) failed for some reason, making this pregnancy a high risk one by default. We would expect a high risk pregnancy to be more closely managed than a low risk pregnancy and for the user to be more alert to any possible problems a well-known, and well documented, limitation of all fetal monitors is that, in the absence of a fetal heart signal, the monitor can pick up on a maternal signal. Best practice, as specified in a number of national safety notices on this subject is to always check the maternal heart rate (pulse) at the start of the trace and record this on the trace. This should be repeated at regular intervals throughout the trace, ensuring that the maternal rate is different from the printed fhr trace and will alert users to the possibility that the fetus may be dead. It is also recommended that a pinard or fetal doppler is used before starting a trace to confirm fetal life and position. As a back-up to this best practice, use of the spo2 sensor (or bp or mecg) will allow the trace to print the maternal heart rate on the same scale as the fetal heart rate trace, making it very obvious if they are the same. It is noted from the trace print out that the spo2 sensor was actually plugged into the monitor during this trace but the absence of

either spo2 values or maternal heart rate suggests that it was not actually applied to the patient. If this sensor had been used, the monitor would have alerted the user to the rates being the same. Before the start of the trace, there are a number of hand written annotations on the trace. One is "bp 116/72", clearly the maternal blood pressure. Below this is an annotation which cannot be read clearly. It is probable that, in line with the above stated best practice, they did check the maternal rate and this was recorded as 92bpm. This being the case, this is within the range of the recorded fetal heart rate trace and could have alerted the clinician to the trace being maternal in origin. There is nothing in this trace to indicate that there is any fault with the fetal monitor. The conclusion based on the limited information provided is that this fetus may have been dead before the trace was started and that the printed trace represents that of the mother. Use of the spo2 sensor would have made this both visually obvious and would have triggered the monitor's cross channel alert risk management file has been reviewed and concluded that the mitigation of the associated risks are appropriate, have been adequately documented and are acceptable, i. E. , warnings have been included in relevant areas. However, we cannot state the problem will not reoccur as further mitigation of these risks is outside our direct control, being driven by local & national guidelines, protocols, training and individual clinician's practices. Similar issue has been reported and this was also concluded as above. Corrective i preventative actions, including field safety corrective actions - none required.

MDR Report Key 4131195
Report Number 1000589001-2014-00004

43) HUNTLEIGH HEALTHCARE LTD, DIAGNOSTICS SONICAID
Model Number FM830ENCORE
Event Date 05/12/2014

Event Description

A (b)(6) gestation fetus who was delivered stillborn (at the end of the trace at 21:27). The baby was very oedematous and swollen and difficult to deliver: there were antenatal complications which were not previously known. The preliminary post mortem report suggests that the fetus had died at least 12 hours prior to delivery i. E. Before the monitoring started. The trace seems to reflect the presence of a fetal heart rate but it does not appear to be the same as the maternal heart rate. Reference mfr report number: 1000589001-2014-00002.

MDR Report Key 3935231
Report Number 1419652-2014-00168

44) PHILIPS MEDICAL SYSTEMS SERIES 50 XM FETAL/MATERNAL MONITOR
Model Number M1350C

Manufacturer Narrative

(b)(4). The customer reported that an infant death occurred while being monitored on a philips device. Due to this request, it is considered that it was unclear for the customer how a fetal heart rate (fhr) could be measured on a fetus which is dead for 2 days. The available information supports that the infant death occurred before the hospital began monitoring using the philips fetal monitor. The device documentation (instructions for use) stresses to confirm fetal life by independent means prior to initiating monitoring. Per the philips response center engineer (rce), there is no indication of fetal life.

Per a philips registered nurse (rn), a review of the provided trace showed that no continuous measurement was used to gather the maternal pulse. Therefore, it was not possible for the device to compare the maternal heart rate (mhr) with the fhr to announce the user of a potential coincidence. Note that the device labeling instructs users to use coincidence detection (ccf) to assure that the measured heart rate is not the maternal heart rate. In addition, please note that the customer has provided 2 trace snippets from 2 different days ((b)(6) 2011), and two different devices for review. Philips will report this incident separately for both involved devices. Philips is in the process of obtaining additional information regarding this incident and the complaint is still under investigation. A final report will be submitted once the investigation is completed.

Event Description

The customer reported that an infant death occurred while being monitored on a philips device.

MDR Report Key 1999792
Report Number 9610816-2011-00091

45) PHILIPS MEDICAL SYSTEMS SERIES 50 XM FETAL/MATERNAL MONITOR

Model Number M1350B

Event Description

The user's report states that they were aware that the baby was not alive, but they were able to obtain hr sounds.

Manufacturer Narrative

(b)(4). The user's report states that they were aware that the baby was not alive, but they were able to obtain hr sounds. This is being reported only because use of the device was coincident with the stillbirth. There is no allegation or indication that use of the device was a factor in the stillbirth. The ability to obtain maternal hr using the us transducer is expected if the users move the transducer until they obtain hr sounds. The device labeling is clear that users are to verify fetal life before monitoring and that users should use the coincidence detection (ccv) feature to assure that the monitoring is not exclusively maternal.

MDR Report Key 1904069
Report Number 9610816-2010-00744

46) PHILIPS MEDICAL SYSTEMS SERIES 50 XM FETAL/MATERNAL MONITOR
Model Number M1350C
Event Description

The customer reported that the device was a factor in the death of an infant during childbirth.

Manufacturer Narrative

The customer reported that the device was a factor in the death of an infant during childbirth. This adverse event is being considered as reportable since it is a death where an allegation has been made that the device was a factor in the death. The customer used a fetal scalp electrode to monitor the fetal ecg and heartrate, but the fetal monitor showed the maternal hr. The midwife did not regard the fetal

scalp ecg or the difference between the fetal scalp electrode and the ultrasound ecg. The midwife did not notice that the fetus died. There is no indication that there was any comparison of the ultrasound fhr to the mother's heart rate as specified in the fetal monitoring instructions for use (ifu). Philips is in the process of obtaining add'l info regarding this event and the complaint is still under investigation. A final report will be submitted once the investigation is completed.

MDR Report Key 1545367
Report Number 9610816-2009-00207

47) PHILIPS MEDICAL SYSTEMS SERIES 50 XM FETAL/MATERNAL MONITOR

Event Description

The customer reported that the fetal monitor on a pregnant pt was giving fhr (fetal heart rate) readings on a pt who had already expired.

Manufacturer Narrative

The customer reported that the fetal monitor on a pregnant pt was giving fhr (fetal heart rate) readings on a pt who had already expired. Philips has explained that in certain cases, the nurse confuses the mhr (mother's heart rate) for fhr, which leads to monitoring of the mhr, while the fetus could be dead. The ifu for this device includes methods for verification of fetal viability when monitoring and for cross-channel verification to assure that the fetus is being monitored. Philips has no indication that these users utilized either of these approaches. The hospital tested the device and found it to be functioning as intended/specified. There is no indication that this issue could be difficult to detect. Additionally, the available information from this report does not support that this issue represents a systemic, design, or labeling problem. The product remains at the customer site. No further investigation or action is warranted.

MDR Report Key 1406028
Report Number 9610816-2009-00051
