# Supplementary material for: Unrecognized maternal heart rate artefact in cases of perinatal mortality reported to the United States Food and Drug Administration from 2009 to 2019: a critical patient safety issue
Source: BMC Pregnancy Childbirth. 2019 Dec 16;19:501. doi: 10.1186/s12884-019-2660-5 (PMC6915916; doi:10.1186/s12884-019-2660-5)
Supplement: Supplementary file 3 — Additional file 3. 14 cases of intrapartum fetal death or neonatal death involving probable maternal heart rate artefact, reported to the US FDA from March 31, 2009 to March 31, 2019 [file 12884_2019_2660_MOESM3_ESM.pdf]

# Additional file 3: 14 cases of intrapartum fetal death or neonatal death involving probable maternal heart rate artefact, reported to the US FDA from March 31, 2009 to March 31, 2019 (Note: all event types for reports listed here classified by the FDA as "death")

NOTE:

Highlighted in yellow: sections which led to classification of the report as probably involving MHRA.

Highlighted in green: sections summarizing the event.

**1) PHILIPS MEDICAL SYSTEMS AVALON FM30 FETAL MONITOR**

Model Number M2703A
Event Date 02/03/2019

Manufacturer Narrative

A follow up report will be submitted once the investigation is complete. Serial number not provided at time of report.

Event Description

The customer reported the death of a newborn after an emergency c-section and 19 minutes of attempted resuscitation. According to the customer (biomedical engineer) and caregivers, artifacts between the mother¿s pulse and the fetus' pulse rate were identified. But they think the device worked properly.

MDR Report Key8331468
Report Number9610816-2019-00044

**2) PHILIPS MEDICAL SYSTEMS AVALON FM50 FETAL MONITOR PERINATAL MONITORING SYSTEM**

Model Number M2705A
Event Date 02/14/2018

Event Description

The customer reported a patient monitoring issue. The customer reported an incident with ¿undesirable results¿. The device was used for monitoring at the time of the alleged malfunction. An incident with ¿undesirable results¿ was reported. The customer did not provide any patient information, although the customer service manager tried to obtain further patient details.

Manufacturer Narrative

The actual monitor used in the incident was checked by a field service engineer (fse) onsite. The device successfully passed the performance verification and electrical safety tests. No issue with the device was found by the fse. The provided trace of the incident was evaluated by product support engineering (pse). The fetal heart rate (fhr) was derived from a cableless (cl) ultrasound (us) transducer. From a technical viewpoint, the derivation of the us signal was excellent, although at around 20:24, the us transducer did not record a signal, likely due to bad positioning of the transducer. At 20:28, the spo2 sensor was removed and the maternal pulse was derived by the cl toco mp transducer. This signal was lost intermittently. From 20:31 to 20:32, the trace shows movement artifacts. Between 20:32 to 20:39, there is no sufficient signal from the mother to allow coincidence detection. A reliable second pulse or heart rate source is required to perform the cross channel verification. From 20:39 onwards, the pulse was again derived by a spo2 sensor. The device issued coincidence alerts at 20:19, 20:22, 20:28, and 20:30 as intended by design. No technical malfunction was observed by pse. The trace was also clinically assessed by a philips physician and an external advisory midwife. They observed that in general the trace shows no accelerations and oscillations with limited undulations. In combination with the decelerations, this pattern presents a suspicious trace. The trace shows deceleration with consecutive loss of fhr baseline. At 20:35, the us transducer probably recorded the maternal pulse source instead of the fetal heart rate. In those cases, the maternal pulse will be shown in fhr trace on the print out. However, because between 20:30 and 20:39, the maternal pulse trace was being lost intermittently, there was not any second pulse or heart rate source at that time; therefore, a cross channel verification could not be performed. At 20:39, it appears that the us transducer switched back to the fetus. At 20:43, a fetal deceleration can be seen. The trace for the fetal signal ended at 20:45; possibly the transducer was removed from the mother¿s belly. The maternal pulse continued to be measured by the spo2 sensor until 21:00. The complete trace ended at approx. 20:57. A comprehensive trace analysis was not possible in this case as it requires additional data, such as therapy provided, patient history, etc. , which was not available in this case. The device worked as designed. No malfunction could be identified based on the provided information. The device remains at the customer site. The customer was informed via customer letter abut the outcome of the investigation. No further investigation or action is warranted.

MDR Report Key7376854
Report Number9610816-2018-00089

**3) PHILIPS MEDICAL SYSTEMS AVALON FM50 FETAL MONITOR PERINATAL MONITORING SYSTEM**

Model Number M2705A
Event Date 11/20/2017

Event Description

The customer called for application support to pull data from a monitor to see if alarms were visible and acknowledged. The customer reported that they had an adverse outcome where there was a concern with regard to heart rate coincidence between the fetal and the maternal heart rate. The device was used for monitoring at the time of the alleged malfunction. The customer stated that there was a delivery with an adverse outcome. No further details about the adverse event were made available by the customer.

Manufacturer Narrative

The issue was evaluated by the clinical specialist (cs) who checked whether the monitor's alarm configuration was set up as discussed with the customer during installation. The cs confirmed that all alarms were enabled correctly and the alarm pause mode was disabled. The cs stated that the trace of the particular adverse event showed question marks indicating the coincidence alarms (as intended when there is a coincidence between the measurements of fetal and maternal heart rate). Despite requested by the cs, the customer did not want to provide the trace for further evaluation by philips. Hence, no further investigation was possible. The cs confirmed that the alarm configuration was set correctly and that the trace of the particular adverse event showed question marks indicating the coincidence alarms. The fse performed a functional check of the monitor at the customer site and confirmed that the monitor worked as specified during testing. The product remains at the customer site. The device worked as intended and no malfunction of the device occurred. The alarm configuration was set correctly and the trace of the particular adverse event showed question marks indicating the coincidence alarms. No further investigation or action is warranted.

MDR Report Key7063811
Report Number9610816-2017-00373

**4) PHILIPS MEDICAL SYSTEMS AVALON FM30 FETAL MONITOR**
Model Number M2703A
Event Date 11/13/2017

Event Description

The customer reported that the (b)(6) monitor did not warn clearly enough of a coincidence and the printout was misleading or unclear. A newborn died when the device was used for monitoring during delivery.

Manufacturer Narrative

The customer stated that the warnings for a questionable fetal heart rate (fhr) were unclear to him, however, he suspected that the maternal heart rate (mhr) had been measured instead of the fhr. The customer complained that the monitor behavior is not clear and not sufficiently described in the instructions for use (ifu). According to the customer¿s problem description, the issue happened in the night from (b)(6) 2017. However, it was then determined based on the provided cardiotocograph (ctg) printout that the delivery took place in the night from (b)(6) 2017. The baby died on (b)(6) 2017. The service distributor was onsite to evaluate the reported issue and confirmed that no malfunction of the device was identified. Nevertheless, the biomedical engineer of the hospital sent the monitor to the philips factory for an additional evaluation. The returned device was checked by product support engineering (pse) who confirmed that the monitor successfully passed the final test. The logs of the

monitor showed coincidence alarms at the time of the reported incident which were silenced manually by a user. By silencing alarms, the user acknowledges all active alarms by switching off audible alarm indicators. Pse confirmed that the device showed no malfunction and worked as specified. The customer was instructed about the intended functionality which is considered as all that is warranted for this issue. The product remains at the customer site and is still in use in the labor and delivery ward. Additionally, the available information from this report does not support that this failure represents a systemic, design, or labeling problem. No further investigation or action is warranted.

**5) PHILIPS MEDICAL SYSTEMS AVALON FM20 FETAL MONITOR**
Model Number M2702A
Event Date 08/13/2017

Manufacturer Narrative

During the birth on (b)(6), a drop of the fhr was seen at 14:40. The baby was born at 14:59 with an apgar score of 2, measured at 1 minute and 5 minutes after birth. The apgar score is a method to quickly summarize the health of newborn children. The baby passed away 3 days after birth due to hypoxic ischemic encephalopathy (hie). The customer provided the trace for the adverse event. The software version of the device is f. 01. 55. The trace it self was printed from a surveillance system, thus no information about the transducer and used monitor was available on the trace. The patient was monitored with a toco transducer and an ultrasound transducer. The maternal heart rate was not monitored, thus no coincidence notation between a maternal heart rate and a fetal heart rate was possible for instances when the ultrasound transducer detected the maternal pulse (e. G. From the aorta abdominalis) instead the fetal heart rate. The trace has been evaluated by a philips physician and an external midwife. They stated that the trace starts with showing signs of compromised fetal well-being through low variability. At 13:36 the fetal trace shows additional decelerations as another sign for clinical deterioration. At 14:06 the fetal trace changes again. The trace shows less decelerations and normal variability. The fhr did not react to contractions any more. This would be an unlikely, sudden clinical improvement of the fetus and is probably caused by the ultrasound switching to the prominent pulse source of the mother. The fetal heart movements are probably too weak or gone from that moment onwards. The device was tested by an engineer, no malfunction could be identified. The problem was solved by instructing the customer which is considered as all that is warranted for this issue. The product remains at the customer site and is used in the labor and delivery ward. Additionally, the available information from this report does not support that this failure represents a systemic, design, or labeling problem. No further investigation or action is warranted.

Event Description

The customer is questioning if the maternal heart rate (mhr) can be recorded as the fetal heart rate (fhr). There was a neonatal patient death reported 3 days after the delivery. The device was used for monitoring at the time of the alleged malfunction.

MDR Report Key6951421
Report Number9610816-2017-00333

**6) PHILIPS MEDICAL SYSTEMS AVALON FM50 FETAL MONITOR PERINATAL MONITORING SYSTEM**
Model Number M2705A
Event Date 05/26/2017

Event Description

The customer reported that a philips fm50 fetal monitor was in use during a critical incident that resulted in an fetal death. The device was used for monitoring at the time of the alleged malfunction.

Manufacturer Narrative

The customer stated there was an unexpected c-section due to the baby's failure to descend on (b)(6) 2017. The approximate duration of the birth was from 13:50 to 17:30. The customer did a c-section and thought everything was well, however, upon delivery the baby was deceased. The fm50 was removed from service by the hospital's clinical engineering department, and was returned to the factory together with the following devices: - device type : m2705a avalon fetal monitor fm50, serial number (sn): (b)(4) with fw revision: a. 06. 31. - device type: m2736a avalon us transducer, serial number: (b)(4), manufactured may 2016. - device type: m2734b avalon toco mp transducer, serial number: (b)(4), manufactured march 2017. The product support engineer (pse) conducted a performance test on the returned equipment. No trouble was found, the devices were fully operational and working as specified. The alarm review (in service mode) was checked on the monitor. A general test on the alarm functionality was performed, the alarming worked as specified. As the monitor was used after the incident, the alarms and stored data for the incident dated (b)(6) 2017, were not available anymore due to limited storage of traces and alarm history. The customer also provided the traces of the reported incident. The traces were reviewed by the product support engineer (pse) and an external senior midwife working closely with philips. During the evaluation of the traces, it was noted that the avalon toco mp transducer listed on the traces was not the same transducer that had been returned to the factory for evaluation (sn: (b)(4) on trace but sn: (b)(4) returned). Both pse and midwife observed that the cardiotocography is clinically conspicuous from the beginning. Restricted oscillation could be observed. The heart rate curve of the fetus is not fluctuating around the baseline when labor pain occurs. From 16:04 onwards, there is no safe recording of the child anymore. The mother was measured with the ultrasound transducer by mistake although the transducer actually should pick up the fetal heart rate.

Coincidence alarms were reported correctly and appear on the traces regularly and repetitively. There are no indications for a malfunction of the device in the recording. The customer additionally sent a second trace of another examination from the monitor. This trace also shows multiple coincidence alarms between the fetal heart rate and the maternal pulse. The trace shows that the device was working as specified during this examination as well. The avalon series fetal monitors utilize ultrasound technology to measure the fetal heart rate non-invasively. It is well documented in the avalon instructions for use (ifu), that phenomena/artifacts such as halving or doubling of the fetal heart rate, or switching between maternal and fetal heart rate can occur when using this method. The equipment was sent back to the customer, 2 of 3 involved devices have been evaluated. No trouble could be found with the evaluated devices and traces. The problem was likely caused by insufficient knowledge of the functionality, and the customer was instructed accordingly. The products remain at the customer site.

MDR Report Key6633726
Report Number9610816-2017-00181

**7)PHILIPS MEDICAL SYSTEMS AVALON FM50 FETAL MONITOR PERINATAL MONITORING SYSTEM**
Model Number M2705A
Event Date 03/30/2015

Event Description

The customer reported "using m2705a avalon fm50 fetal monitor with m2734b avalon toco mp transducer, were getting coincidence alarm due to maternal pulse the same or close to fetal heart rate hr, using m2736a avalon us transducer. The baby died at birth".

Manufacturer Narrative

The research and development department (r&d) reviewed the provided trace copy submitted to us and the provided response and found from a technical point of view, there is no indication of a product malfunction. Result: from a technical point of view the tracings look correct. There is no indication of equipment malfunction. Investigation summary: a philips field service employee retrieved the log and configuration files from the device and provided these to r&d for analysis. R&d confirms that several device configuration settings were adapted by you to meet your needs. It was noted that the acoustical ccv inop warning was enabled to sound immediately with minimum volume of ¿4¿. The error log did not contain entries related to any device malfunctions. The following equipment was involved as documented on the header of the fetal trace (collectively, the ¿equipment¿): m2705a avalon fm50 fetal/maternal monitor, serial number: (b)(4), software revision j. 30. 59 (the ¿monitor¿). M2736a us transducer, serial number: (b)(4), software revision a.06.31 (the ¿us¿). M2734b toco mp transducer, serial number: (b)(4), software revision a. 06. 31 (the ¿toco mp¿) the trace recording, log and configuration files from the device were examined by the philips research and development department (¿r&d¿) and the results were as follows. R&d confirms that all of the equipment functioned as specified and that no malfunctions were identified. Fetal trace analysis: r&d has reviewed and analyzed the copy of the fetal trace in detail. The trace starts on (b)(6) 2015, 23:25, and ends on (b)(6) 2015, 00:52. Summary: the toco mp correctly documented the maternal pulse rate which went up above the fetal heart rate during pushing (2nd stage of labor). During contractions the ultrasound transducer picked up a maternal signal (¿maternal switching¿ artifact). This was confirmed by the spo2 sensor applied shortly after midnight. Later the ultrasound transducer continuously recorded a maternal pulse rate, as indicated repetitively by the question marks on the trace (cross-channel verification = ccv). Details: paper grid and page numbers are not visible on the trace copy provided (brightness/contrast too high). A separate copy of the trace header showed equipment information (serial number, software revision) as documented above, printed at 21:21. International paper scaling with paper speed 3 cm/min has been used. Page 1 of the trace copy starting around 23:25: the records show that the us and toco mp transducers were plugged in, but only the us trace is visible on the trace copy. Movement bars are printed (fetal movement profile = fmp). In the 2nd stage of labour these movements mostly are of maternal origin. Page 2: the toco and mp recordings are starting. Between contractions the maternal

pulse is 20 to 30 bpm below the fetal heart rate. During uterine contractions (pushing) the maternal pulse goes up above the fetal heart rate and the ultrasound temporarily switches to the maternal rate, indicated by the toco mp trace. Pages 3 to 5 starting at around 23:39: uterine contractions with pushing continue with strong maternal pulse rate accelerations. A uterine contraction may result in moving the fetal heart temporarily out of the us beam, and the signal from a maternal vessel can be picked up during this time. The cross-channel verification (which is indicated by ccv question marks on screen and on top of the recording) correctly indicated that the us and toco mp picked up a signal from the same source, i. E. Maternal. Between contractions the ultrasound returns to the fetal signal.

Page 5: after 23:53 the ultrasound almost continuously records a maternal signal. Ccv warning is given repeatedly. Page 6: at 00:02 the records indicate that a spo2 sensor has also been applied to the patient which automatically replaced the maternal pulse trace from toco mp. The trace patterns shown by the spo2 sensor are consistent with the trace patterns previously recorded by toco mp. This confirms that toco mp has correctly picked up the maternal and not the fetal pulse rate. Note: toco mp can pick up a fetal pulse rate only if a fetal artery is extremely close to the optical sensors of the transducer.

Pages 7 to 13: the fetal monitor correctly gave ccv warning as documented on the paper. During a heart rate coincidence condition the affected heart rates are marked on the fetal monitor screen with a question mark. In addition an acoustical inop is given (software revision j. 30). Pages 9, 11, 13: ccv warning is given although only the ultrasound trace is printed: spo2 had signal loss (not applied to the patient?) and the maternal pulse trace (toco mp) on the recorder had been manually switched off. The ccv feature continues to work even if the mp trace recording is disabled. Accordingly, the tests and analysis performed by r&d confirm that the device worked as specified. There is nothing in the records to indicate any device or equipment malfunction. The baby died at birth. The customer would like to know if m2705a avalon fm50 fetal monitor s/n: (b)(4) worked properly. The m2705a avalon fm50 fetal monitor s/n: (b)(4) was used at the time of the stillbirth for monitoring. After fetal trace analysis per r&d, no indication of any malfunction was found. The m2705a avalon fm50 fetal monitor s/n: (b)(4) remain at the customer site. There is no indication of any m2705a avalon fm50 fetal monitor malfunction.

The device was not contributory to the reported stillbirth. The cause of the baby's death is unknown. No further investigation or action is warranted. (b)(4).

MDR Report Key4710453
Report Number9610816-2015-00081

**8)PHILIPS MEDICAL SYSTEMS AVALON FM50 FETAL MONITOR**
Model Number M2705A
Event Date 10/14/2014

Manufacturer Narrative

(b)(4). A follow up report will be submitted after philips obtains more information concerning this event.

Event Description

The customer reported that a female who has had one pregnancy and no live births presented in active labor ((b)(6)) to labor and delivery with contractions. During the second stage of labor, an emergent c-section was required due to fetal bradycardia that was not apparent on the electronic fetal monitor until an internal fetal scalp electrode was placed. At the time the bradycardia was discovered, the c-section was performed. The baby had a triple nuchal cord, born with no detectible heart rate and neonatal resuscitation program was initiated. The pt was transported to the nicu in critical condition.

The infant was resuscitated post-partum, and transferred to (b)(6) where they determined the infant had no brain activity thus transferred back. This was a post-term infant with (b)(6) complete weeks of gestation. The baby was delivered asystolic, and required full resuscitation including intubation, positive pressure ventilation (ppv), chest compression, multiple epinephrine doses and central line placement. Her apgars where 0, 0, and 0 at 1, 5 and 10minutes. Heart rate was obtained approximately 13 minutes into resuscitation. Severe hypoxic ischemic encephalopathy; the family with drew life support and infant expired two days later.

MDR Report Key4351506
Report Number9610816-2014-00316

**9) PHILIPS MEDICAL SYSTEMS AVALON FM50 FETAL MONITOR**
Model Number M2705A
Event Date 07/31/2013

Manufacturer Narrative

(b)(4). A follow-up report will be submitted after philips obtains more information concerning this event.

Event Description

The customer reported a fetal death while monitoring with an avalon fm50 fetal monitor. The customer requested that an audible inop for the coincidence detection alerts be introduced.

MDR Report Key3299684
Report Number9610816-2013-00173

**10)PHILIPS MEDICAL SYSTEMS AVALON FM30 FETAL MONITOR**
Model Number M2703A

Event Description

The customer reported that a baby died after being monitored by a philips device.

Manufacturer Narrative

(b)(4). The customer reported that a baby death occurred after being monitored by a philips device. This is being reported only because a philips device was in use on a baby who died. Based on the current, available information, the maternal heart rate (mhr) increased and coincided with the fetal heart rate (fhr), however, the fetal monitor showed/printed question marks. According to the statement from the head physician, the question marks were either ignored or not correctly interpreted due to human error. The baby suffocated during the birth. There is no indication of any malfunction of the (b)(6) avalon fm30. Philips is in the process of obtaining additional information regarding this incident and the

complaint is still under investigation. A final report will be submitted once the investigation is completed.

MDR Report Key 2012314
Report Number 9610816-2011-00116

**11)PHILIPS MEDICAL SYSTEMS AVALON FM50 FETAL MONITOR**
Model Number M2705A
Event Date 01/07/2010

Event Description

On february 12, 2010, philips received a medwatch report stating that the baby died one day after the delivery.

Manufacturer Narrative

On february 12, 2010, philips received a medwatch stating that the baby died one day after the delivery. The customer made it clear in the medwatch that there have been difficulties distinguishing between the baby's and the maternal hr. And therefore, a c-section was performed. The customer made it clear that the clinicians were aware of the issue and made no allegation or indication that the device contributed to the death. In addition, there is no allegation of a product malfunction. Philips is in the process of obtaining additional info regarding this event and the complaint is still under investigation. A final report will be submitted once the investigation is completed. (b) (4).

MDR Report Key1622600
Report Number9610816-2010-00042

**12) PHILIPS HEALTHCARE, INC. PHILIPS AVALON FM50 FETAL MONITOR**
Model Number FM 20
Event Date 11/30/2009

Event Description

A pt presenting to labor and delivery in labor. At 41 week gestation and history of prior planned c-section for twins. Patient placed on fm20 philips avalon. Monitor was erratic with tracings and at one point, it was difficult to distinguish mom and baby's heart rate on the tracing, but the digital readout was 30 beats apart. Ultrasound showed no heart beat. Emergency c-section was performed and the infant had an apgar of 0,0. The infant was resuscitated and transferred to facility with higher level of care in nicu. The baby did not survive, dying the next day.

MDR Report Key1579412
Report Number1579412

**13)PHILIPS MEDICAL SYSTEMS ULTRASOUND TRANSDUCER FOR USE WITH FM20/FM30 AVALON**Model Number M2736A
Event Date 01/24/2012

Event Description

The customer sent an email query regarding a field action in 2009 for philips avalon fetal monitors. This was in relation to a case regarding a baby who died in (b)(6) hospital in 2012. A newborn died when the device was used for monitoring during delivery at (b)(6) 2012. The incident took place in the hospital delivery room.

Manufacturer Narrative

A baby died on (b)(6) 2012. The incident was not reported to philips at that time. The investigation determined that the customer requested assistance to clarify questions related to the field safety notice from 2009 concerning the ultrasound (us) transducer signal ( i. E. Device detecting the fetal heart rate (fhr) ). This complaint is registered on the us transducer as the doppler echoes are processed by the mainboard within the ultrasound transducer by an auto-correlation algorithm to determine fetal heart rate (fhr). The signal processing of the fhr is done by the firmware (software) on the transducer mainboard. The fhr is only reported on the monitor¿s numeric display and on the recorded trace. The full traces of the incident have been provided by the (b)(4) to philips for an evaluation by product support engineering (pse) and a clinician. Pse stated that the traces show that the customer used multiple devices during that patient monitoring episode. A philips physician and an external midwife assessed the traces and concluded the following: the trace shows fetal distress starting at 06:32 am. From that moment onwards, the fhr trace rarely shows signals from the fetus, and is instead almost exclusively showing a maternal signal. This phenomenon is well-known and inherent to the fetal monitor¿s ultrasound technology. Therefore, the fetal monitor is designed to compare a known maternal signal (e. G. Pulse measured by an spo2 finger sensor) with the ultrasound signal. This coincidence analysis is continuously done by the monitor and alerts the user in case of a coincidence. Here, the coincidence analysis was only intermittently possible because the maternal spo2 probe was not used continuously after epidural anesthesia was started. However, during those periods when the spo2 sensor was  applied to the mother, the fetal monitor issued multiple coincidence alerts consistent with its design and labeling. The strips provided to philips show no indication of device malfunction. The investigation reveals that there is no relation between the death on (b)(6) 2012 and the field actions from 2009. The traces provided to philips show no indication of a device malfunction. The products remain at the customer site. The provided information shows that all avalon devices involved in the incident had the latest firmware on (b)(6) 2012. This complaint does not represent a product/part failure. No further investigation or action is warranted.

MDR Report Key7233465
Report Number9610816-2018-00035

**14) WIPRO GE HEALTHCARE PRIVATE LTD COROMETRICS MONITOR PERINATAL MONITORING SYSTEM**
Model Number 259 CX-C
Event Date 08/05/2012

Event Description

Ge healthcare has received notification of a death of a fetus.

Manufacturer Narrative

The legal complaint that general electric company received alleges the following: "the corometrics monitor made it appear to the healthcare providers that the fetal heart rate was being monitored throughout labor and was normal. " "however, at some point hours before delivery, his fetal heart rate became distressed. Instead of picking up this distress, the corometrics fetal heart monitor made a smooth transition to the maternal heart rate, confusing the healthcare providers into believing the baby's heart was fine. " ge healthcare's investigation is ongoing. A follow up report will be submitted once the investigation has been completed.

Manufacturer Narrative

No information has been provided to ge healthcare by the hospital on the status of the unit. At this time, it is not known if the unit was taken out of service or if it continues to be used with patients. No further details about the alleged device or the event have been provided to ge healthcare. There are no service records for the device in the ge healthcare database. Therefore, without sufficient information about the event, or the evaluation of the alleged device, it is not possible to determine the root cause of the alleged issue.

Manufacturer Narrative

The following information was obtained by ge healthcare through the legal proceedings related to this case. Multiple clinical signs presented that indicated fetal distress during the monitoring session to which the ob team did not take appropriate actions: low fetal heart rate variability- a healthy fetus has high heart rate variability. Maternal heart rate was very close to fetal heart rate; the strip chart indicated the maternal heart rate and fetal heart rate actually overlapped 5 times during the monitoring session, as shown by the hbc indication- a healthy fetal heart rate is typically higher than the maternal heart rate. Fetal heart rate accelerated during contractions- a healthy fetus would have heart rate decelerations during maternal contractions. Multiple maternal parameters to indicate the mother was at high risk for a complicated birthing process, i. E. She was overweight, tachycardic, and feverish. The ob team attempted to use a fetal scalp electrode (fse) to obtain a fetal heart rate directly on four occasions with two different electrodes. They observed no good signal from the fse attempts and concluded the electrodes were defective, instead of concluding there was a problem with the fetus. One of the obstetricians communicated they do not look at the monitor strip chart at all. Another member of the ob team communicated they didn't know about overlapping heartrates and, therefore, did not understand the indications. The obstetricians communicated they did not read the monitor user's manual and did not understand heartbeat coincidence. The obstetricians applied, removed, and then applied spo2 again. When spo2 was applied and indicated a maternal heart rate that was overlapping with the fetal heart rate, appropriate actions were not taken. The obstetricians did not consider the two heart beats were both from the mother and that the fetal heart beat was not being detected. Ge healthcare provided training on the device which was attended by hospital staff several months prior to the incident. The hospital is responsible for ensuring their clinicians are properly trained on the device through personnel turnover cycles, etc. The ge training was provided one time only and was not purchased again for new clinicians that did not receive the original training. It was concluded that the root cause was user error interpreting the clinical situation of the mother and the fetus.

MDR Report Key3369763
Report Number9617277-2013-00001
