# Supplementary material for: Unrecognized maternal heart rate artefact in cases of perinatal mortality reported to the United States Food and Drug Administration from 2009 to 2019: a critical patient safety issue
Source: BMC Pregnancy Childbirth. 2019 Dec 16;19:501. doi: 10.1186/s12884-019-2660-5 (PMC6915916; doi:10.1186/s12884-019-2660-5)
Supplement: Supplementary file 4 — Additional file 4. Clinical Summary: Diagnosis and Management of Cardiotocography Artefact [file 12884_2019_2660_MOESM4_ESM.pdf]

Additional file 4.
Clinical Summary: Diagnosis and Management of Cardiotocography Artefact

Types of cardiotocography artefact:

Maternal Heart Rate Artefact (MHRA): occurs when the maternal heart rate is captured as the input and the electronic fetal monitor mistakenly outputs this signal as the fetal heart rate.

Fetal Heart Rate Artefact (FHRA):
FHRA Type 1– FHR may be doubled or halved or subject to other signal processing errors.
FHRA Type 2 – In multiple gestation, FHR of an unintended fetus (eg. Twin B) is displayed instead of that of the intended fetus (eg. Twin A).

Signal ambiguity = MHRA and/or FHRA Type 2. (common element = capturing heart rate from the wrong source, i.e. maternal instead of fetal or unintended fetus in multiple gestation)

Strategies to detect and manage Fetal Heart Rate Monitoring Artefacts:

Consider continuously monitoring maternal pulse whenever cardiotocography is used, particularly in 2nd stage.

Use **Signal Ambiguity Detection Technologies** detection technologies, if available: for example, Cross-channel verification (Philips) or Coincidence detection (General Electric).

Signal Ambiguity Detection Technologies involve:
-continuously monitoring of the maternal heart rate along with the fetal heart rate
-simultaneous display of the maternal heart rate along with the fetal heart rate on the monitor and tracing paper
-alarms which are emitted when the maternal and fetal heart rate signals are close enough that it raises concern they may be from the same source (in effect, both signals are maternal and/or both signals are from the same fetus in multiple gestation)

Know your coincidence alarms (Philips – Question mark symbol, General Electric – Overlapping hearts).

Know if coincidence alarms will be displayed on 3rd party fetal surveillance technology, if using. They may not be visible on a 3rd party fetal surveillance display.

Use best clinical acumen to detect MHRA and FHRA Type 2, especially if Signal Ambiguity Detection Technology is not available.

Suspect MHRA clinically when there appears to be:

-sudden apparent sustained improvement of an abnormal tracing to a normal pattern
-sudden apparent change from a poor to high quality tracing
- accelerations with pushing or contractions
-marked accelerations and decelerations
-sudden change to a new baseline
-fluctuation in the baseline
-fluctuation in the character of the tracing
-doubling/halving of the rate
-confusing pattern
Especially if the above occurs:
-after a position change
-after a gap in monitoring
-after an epidural
-in the second stage with pushing

Suspect FHRA Type 2 clinically when:

-the supposedly different fetal heart rates are close together in a multiple gestation

Suspect FHRA Type 1 clinically when there appears to be:

-doubling/halving of the rate
-marked accelerations and decelerations
-sudden change to a new baseline
-fluctuation in the baseline
-fluctuation in the character of the tracing
-confusing pattern

Management of suspected CTG artefact.

Respond appropriately to coincidence alarms and/or other signs of artefact in a rapidly escalating fashion:
-Reposition transducers.
-Consider fetal scalp electrode (remember artefact may still occur with a fetal scalp electrode).
-Consider direct visualization of fetal heart rate with bedside ultrasound (gold standard)

Know your fetal monitor.

Ensure you are familiar with the sections of the fetal monitor's user manual which discuss maternal heart rate artefact diagnosis and management, especially for fetal monitors employing signal ambiguity detection technologies. If you are in a leadership position, consider organizing periodic training for all staff. Ensure all new staff are trained in how to use the CTG monitor in your hospital.

What's new for experienced clinicians?

The introduction of autocorrelation algorithms with modern CTG monitors, while generally improving the fetal heart rate signal, may lead to more subtle and difficult to detect maternal heart rate artefact and type 2 fetal heart rate artefact (also known as signal ambiguity) with smooth transitions rather than breaks in the tracing. Therefore, training with signal ambiguity detection technologies which simultaneously monitor the maternal and fetal heart rates and/or multiple fetal heart rates in multiple gestations is important, even for experienced clinicians.
